# Precision and Accuracy of a Direct-Reading Miniaturized Monitor in PM_2.5_ Exposure Assessment

**DOI:** 10.3390/s18093089

**Published:** 2018-09-13

**Authors:** Francesca Borghi, Andrea Spinazzè, Davide Campagnolo, Sabrina Rovelli, Andrea Cattaneo, Domenico M. Cavallo

**Affiliations:** 1 Department of Science and High Technology, Università degli Studi dell’Insubria, via Valleggio 11, 22100 Como, Italy; davide.campagnolo@uninsubria.it (D.C.); sabrina.rovelli@uninsubria.it (S.R.); andrea.cattaneo@uninsubria.it (A.C.); domenico.cavallo@uninsubria.it (D.M.C.); 2 Department of Statistics, Informatics and Applications “G. Parenti”, Università degli Studi di Firenze, viale Morgagni 59, 50134 Firenze, Italy

**Keywords:** exposure assessment, particulate matter, air pollutant, environmental monitoring, low cost sensor, performance evaluation, personal exposure

## Abstract

The aim of this study was to evaluate the precision, accuracy, practicality, and potential uses of a PM_2.5_ miniaturized monitor (MM) in exposure assessment. These monitors (AirBeam, HabitatMap) were compared with the widely used direct-reading particulate matter monitors and a gravimetric reference method for PM_2.5_. Instruments were tested during 20 monitoring sessions that were subdivided in two different seasons to evaluate the performance of sensors across various environmental and meteorological conditions. Measurements were performed at an urban background site in Como, Italy. To evaluate the performance of the instruments, different analyses were conducted on 8-h averaged PM_2.5_ concentrations for comparison between direct-reading monitors and the gravimetric method, and minute-averaged data for comparison between the direct-reading instruments. A linear regression analysis was performed to evaluate whether the two measurement methods, when compared, could be considered comparable and/or mutually predictive. Further, Bland-Altman plots were used to determine whether the methods were characterized by specific biases. Finally, the correlations between the error associated with the direct-reading instruments and the meteorological parameters acquired at the sampling point were investigated. Principal results show a moderate degree of agreement between MMs and the reference method and a bias that increased with an increase in PM_2.5_ concentrations.

## 1. Introduction

Presently, particulate matter (PM) is considered as one of the main air pollutants [1], since several epidemiological and toxicological studies have reported associations between PM and its effects on human health [2,3,4,5]. Thus, air quality monitoring is frequently required by national and international regulations [6,7]. 

The inadequacy of traditional fixed air quality stations in assessing human exposure to PM has emerged in recent years and their main disadvantages are related to: (i) the inability to provide data at high spatial and temporal resolutions—a limitation essential in urban environments [8,9]; (ii) the necessity of technical and logistic infrastructures (power supply, protection structures, etc.) [9,10]; and, (iii) the high cost/high level of maintenance [6,11]. Due to these limitations, several portable monitors have been developed which provide data (i) at high spatial and temporal resolutions; (ii) at individual or personal levels; (iii) characterized by real-time responses [12]; and, (iv) provide air pollutant exposure values for the selected subject. Previous studies have tested several portable monitors through laboratory tests with standard aerosol, outlining that such monitors are generally characterized by a worse performance than reference measurement methods [13,14,15,16,17,18,19]. Nevertheless, only few studies aimed at evaluating the performance of these monitors were conducted in field and in real-world conditions [20,21,22,23,24,25,26,27]. However, studies regarding the evaluation/comparison of miniaturized monitors (MMs) are few. MMs are characterized by several advantages because they are (i) compact; (ii) lightweight; (iii) inexpensive; (iv) energy-efficient; (v) easy to use and portable; and, (vi) are able to provide data at high spatial and temporal resolutions [28,29,30]. Presently, many of these monitors are neither well evaluated in the scientific literature nor compared with reference methods. Therefore, the aim of this study is to evaluate the performance of an MM for direct-reading (real-time) measurement of PM_2.5_ (AirBeam, HabitatMap Inc., Brooklyn, NY, USA; particle sensor: Shinyei PPD60PV—abbreviated ‘AB’). AB was selected among other sensors [28] mainly due to its practicability (as discussed in Section 4.1), since ABs are intended to be used in a future exposure assessment study by the authors. However, presently, scientific articles regarding the use of AB are few: for this reason, it was necessary to deepen the issue of AB’s precision and accuracy and provide further information in this regard.

In particular, only three studies have been conducted to evaluate accuracy, precision, and reliability of such miniaturized and low-cost sensors in field and real-world conditions [31,32,33] (Table 1). Mukherjee et al. [31] evaluated the performance of the AB over a 12-week period in Cuyama Valley (California, USA). Contrariwise, Sousan et al. [32] evaluated performances of different consumer air quality monitors (including AB) in laboratory tests and over a wide range of mass concentrations. Finally, the multi-year CAIRSENSE project [33] tested different instruments in the field. 

It should be noted that studies that evaluated other types of MMs or measurement devices based on the Shinyei PPD60PV sensor [34,35] are not reported in Table 1, since the aim of this study is to specifically evaluate the AB monitor and its potential applicability for exposure assessment studies, wherein the performance depends not only on the kind of sensor but also on other factors, such as the type of hardware and software system, as well as calibration factors and correction algorithms used.

## 2. Materials and Methods

### 2.1. Study Design

This study consisted of a field campaign carried out to evaluate the performances of co-located MMs in comparison with a reference (gravimetric) method for PM_2.5_ and with other widely used portable PM monitors.

The campaign was performed during two different periods (warm period: 24 July 2017–8 August 2017; cold period: 10 January 2018–7 February 2018) at an urban background site described elsewhere [20,36]. An urban background site was chosen according to the Guidelines for Air Quality Monitoring Network provided by the Agency for Environmental Protection and Technical Services [37] to acquire data representative of the average pollution levels in the study area. Moreover, measurements were performed across different periods of the year to evaluate the performance of sensors under different meteorological and environmental conditions. In each season, 8-h long (8 AM to 4 PM) monitoring sessions (N = 10) were conducted.

The sampling equipment was placed in a dedicated sampling station, which is approximately 1.5 m above the ground, far from obstructions, walls, and pollution sources.

All of the instruments were positioned at about 20 cm from each other to avoid possible interferences. Clocks for all the instruments were synchronized at the first measurement session and were checked at the beginning of each 8-h sampling (Figure S1).

To ensure that quality-controlled data were collected, all of the direct-reading instruments were operated following the manufacturer guidelines and using the factory-supplied calibration factors. Further, before and after each monitoring session, a zero calibration was performed for Optical Particle Counters (OPC) and Aerocet with appropriate HEPA absolute filter (rated at 99.96% removal efficiency for 0.45 mm particles). During monitoring, the functionality of the instruments was checked hourly to avoid malfunctions or data loss. Immediately before the study, all of the instruments were checked by factory services to verify their compliance with the product specifications.

### 2.2. Instruments: PM

To assess the performance of the MM, direct-reading instruments and a reference filter-based technique were selected for comparison.

Specifically, two Optical Particle Counters were used as direct-reading devices, and specifically a Handheld 3016 IAQ (abbreviated “OPC”—Lighthouse Worldwide Solutions, Fremont, CA, USA; counting efficiency: 50% at 0.3 nm; 100% for particles >0.45 nm;) and an Aerocet-831 (abbreviated “Aerocet”—Aerosol Mass Monitor, Met One Instruments, Inc., Grants Pass, OR, USA; accuracy ±10% to calibration aerosol). Both of the instruments classify PM into different fractions, including PM_2.5_, and they are based on the principle of light scattering while using an active sampling mode with a flow rate of 2.83 L/min.

The filter-based instrument for the gravimetric determination of PM_2.5_ (used as reference method in this study) was an EPA Well Impactor Ninety–Six (“EPA WINS”; Federal Reference Method for PM_2.5_) which operates using a sampling pump (Digit ISO, Zambelli, Milan, Italy) at a flow rate of 16.7 L·min^−1^. Particles were collected on 47 mm glass fiber filters (Whatman GF/D glass microfiber filters) and mass concentrations were determined via gravimetric analysis following a standard reference method [38,39]. The weighing filters were conditioned in a controlled environment (temperature: 20.0 ± 1.0 °C; relative humidity (RH): 50 ± 5%) for a minimum of 24-h following which the filters were weighed, before and after the sampling, with a microbalance (Gibertini Micro1000, Novate, Milan, Italy; readability: 1 µg). An electrical C-shaped ionizer (HAUG GmbH & Co. KG, Leinfelden—Echterdingen, Germany) was used to eliminate electrostatic charges from the filter surface. Two laboratory blanks were also weighed under the same conditions to identify the possible anomalies in the weighing room environment (temperature and humidity variations). To check the accuracy of the microbalance, certified masses of 1 and 100 mg were always weighed at the beginning and at the end of each weighing session, allowing for deviations of ≤3 and 5 µg, respectively, from the true value.

Finally, three ABs (instruments that reflect the MM characteristics reported above) represented the MMs to be evaluated in this study. The sensor is based on an Arduino board and can detect particles ranging from 0.5 to 2.5 µm and PM_2.5_ concentrations up to 400 µg/m^3^ [32,40]. These monitors are characterized by reduced dimensions (10.46 cm × 10.03 cm × 4.62 cm), low weight (198 g), and low costs (about USD 250, according to [31]). The air was drawn through the sensing chamber by means of an internal fan where an LED light source scattered off particles. The light scatter produced was then detected and the instrumental signal was converted to a mass concentration value while using a linear regression model [32]. The acquired data were sent via Bluetooth, approximately once per second, to an open source Android Application (AirCasting Android app, HabitatMap Inc., Brooklyn, New York, NY, USA), from which they can be downloaded [41].

### 2.3. Instruments: Meteorological Data

An external weather station (BABUC-ABC, LSI Lastem, Milan, Italy) was placed at the same sampling point to characterize the meteorological conditions. In particular, temperature (°C), RH (%), atmospheric pressure (hPa), wind intensity (m/s), and wind direction (°) data were acquired. The weather station was programmed with an acquisition rate of 1 min and an elaboration rate of 60 min. The acquired data were processed every hour to provide: (i) hourly averages; (ii) standard deviations (S.D.); (iii) maximum; (iv) minimum; and, (v) time of maximum and minimum values. Hourly mean rainfall data were obtained from the nearest monitoring station of the Regional Agency for Environmental Protection of Lombardy (Como, ARPA—Agenzia Regionale per la Protezione Ambientale—Villa Gallia) located 2.5 km NW from the sampling point.

### 2.4. Statistical Analyses and Data Treatment

Statistical analyses were performed while using SPSS Statistics 20.0 software package (IBM, Armonk, NY, USA). To exclude unrealistic low and high concentration values, all data (except meteorological data averaged for the 1-h period) were truncated below the 1st percentile and above the 99th percentile [3]. A *p*-value lower than 0.05 was considered as statistically significant for all tests. Descriptive statistics were estimated for PM_2.5_ concentration outcomes from all instruments and for meteorological data for the single monitoring sessions, the two seasons, and the entire study period.

The evaluation of the AB by comparison with the reference method (as well as other direct-reading instruments) was carried out using different tests: (i) precision evaluation (evaluation of uncertainty between co-located MMs by means of uncertainty analysis and linear regression, according to the indications summarized by Watson et al. [42]); (ii) comparison with reference gravimetric method (Mann-Whitney test, Spearman’s correlation (rho); regression analysis according to the indications that were summarized by Watson et al. [42]); (iii) evaluation of error trends (Bland-Altman plot method; absolute and relative errors); and, (iv) impact of meteorological variables on measurement errors (multiple linear regression analysis between AB absolute errors and meteorological parameters; only independent variables that were found to be statistically significant in the bivariate correlations were included in each multivariate model).

1-min averaged data were used for comparisons among direct-reading instruments (AB, Aerocet, OPC) while 8-h averaged values were used for comparisons between direct-reading instruments and the gravimetric reference method (EPA WINS). Because of the high strength of the relationships between co-located AB, as described in the Results and Discussion sections, for convenience, the mean of data for all the ABs was used as a new variable for the statistical analyses. Results regarding each AB device (AB1, AB2, and AB3) are reported in the supplementary material.

The uncertainty between couple of ABs was calculated following the guidance that was reported by the EC Working Group [43]. AB data were averaged for 8-h instead of 24-h since the study design was based on a period of 8-h. The uncertainty of AB was calculated from the difference of measure according to Equation (1):

(1) $$\;u_{bs}^{2}=\frac{\sum_{i=1}^{n}{\left(y_{i,1}-y_{i,2\;}\right)}^{2}}{2n}\;$$

Equation (1) Uncertainty formula used in this study. *u*^2^*_bs_* represents the uncertainty; *y_i_*_,1_ and *y_i_*_,2_ represent AB measurements averaged for the entire monitoring session period (8-h); n represents the number of the total measurements considered in the analysis.

Following the guidance report, the uncertainty was determined for the total dataset as well as for the two datasets that were obtained by splitting the entire dataset according to PM_2.5_ concentrations: ≥18 µg/m^3^ and <18 µg/m^3^. Moreover, in this study, the uncertainty was also calculated separately for the summer and winter datasets. According to the guidance report, an uncertainty >2.5 µg/m^3^ must be considered as an indication of unsuitable performance for one or both of the co-located instruments.

Linear regression was used to evaluate the level of agreement between the two methods and the reference method was considered as the independent variable while the method to be tested was the dependent variable. As reported by Watson et al. [42,44], equation parameters (R, slope, and intercept) can be used as indicators of the comparability and/or predictability between the two methods. In particular, the two methods can be classified as comparable and mutually predictable (i.e., the independent and dependent variables are considered interchangeable) if: (i) slope is equal to 1 ± 3 standard error (s.e.); (ii) intercept is equal to 0 ± 3 s.e.; and, (iii) R > 0.9. If R is >0.9 but the slope and intercept criteria are not met, the investigated methods can be considered as comparable but only the dependent variable is predictable from the independent variable. Finally, methods with R < 0.9 are classified as not comparable.

Additionally, Bland-Altman plots were used to evaluate possible error trends [45,46]. In the present study, the plots were based on the entire dataset and reported absolute deviation between measurements and the upper and lower confidence intervals (calculated as the average difference ± 1.96 S.D. of the differences).

## 3. Results

### 3.1. Average PM_2.5_ Levels and Meteorological Parameters

Repeated 8-h monitoring sessions (N = 20 sessions; >150 h) were conducted at the urban background station during summer (July 2017–August 2017) and winter (January 2018–February 2018). A total of 20 filter-based samples (N = 19 valid) and N > 7000 were collected from gravimetric samplers and direct-reading instruments, respectively.

Table 2 and Table 3 present the summary statistics of the PM_2.5_ concentrations and the meteorological data that were acquired from all instruments during the two monitoring periods.

During the warm period, the mean concentration values (mean ± S.D.) of ABs were similar (7.1 ± 4.7; 6.5 ± 4.2, and 6.8 ± 4.8 µg/m^3^, respectively) and comparable to the average OPC concentration (6.6 ± 4.7 µg/m^3^) (Table 2). On average, AB data tended to underestimate PM_2.5_ levels when compared to Aerocet and the reference gravimetric method for PM_2.5_ (12.3 ± 8.9 and 12.5 ± 7.2 µg/m^3^, respectively) (Table 2). During the cold period, the average PM_2.5_ concentrations were equal to 34.9 ± 29.5, 40.8 ± 32.2, and 37.9 ± 28.5 µg/m^3^ for AB1, AB2, and AB3, respectively. Additionally, the AB values were lower than the Aerocet concentrations (50.8 ± 46.5 µg/m^3^) but higher with respect to the average value for EPA WINS (22.8 ± 48.3 µg/m^3^) (Table 2). When considering the entire dataset, maximum PM_2.5_ concentrations were reached during the 18th monitoring session (EPA WINS: 48.3 µg/m^3^; AB1: 98.1 µg/m^3^; AB2: 102.7 µg/m^3^; AB3: 88.0 µg/m^3^), while the lowest values were registered during the first summer monitoring session (EPA WINS: 2.2 µg/m^3^; AB1: 1.3 µg/m^3^; AB2: 1.3 µg/m^3^; AB3: 1.2 µg/m^3^).

The warm period was characterized by low RH (mean: 40.7%) and by high temperature (mean: 29.2 °C; min.: 17.1 °C; max.: 39.7 °C). Typical winter meteorological parameters were found during the cold period. The average RH was equal to 67.8%, while the temperature ranged from −0.9 °C to 14.0 °C (mean: 7.7 °C). The sampling site was characterized by generally low wind speeds (also reported in a previous study carried out in the same area [36]), mainly because of the sampling location (approximately 1.8 km from the banks of Lake Como) and the local topographic scenario (with moraine hills which surrounded the area). During the warm period, the wind intensity was <1.5 m/s in 96% of the cases (and <1 m/s in 62.7% of the cases), while during the cold period, the wind speed was <1.5 in 70.6% of the cases. Wind blew principally from S during summer and from SW during winter (Figures S2 and S3). 

### 3.2. Precision Evaluation: Comparison among AB Copies

As previously stated, linear regression analyses were carried out on the total dataset with 1-min averaged values, and regression parameters were used as indicators of precision of co-located ABs (Table 4).

As reported in Table 4, R^2^ values were always very high (>0.98). Nevertheless, the tested instruments can be classified as comparable but not mutually predictable, because of non-compliance with the slope and intercept criteria with regard to the Watson et al. approach [42,44]. 

Additionally, the absolute error (defined as the difference between tested and reference measurement) and relative error (absolute error divided by reference measurement) between the ABs were evaluated [47]. The mean absolute error between the three ABs was 5.7 µg/m^3^, while the relative error was 9% (Table S1).

Subsequently, the uncertainty between pairs of co-located AB was calculated following the guidance for demonstration of equivalence [43] and it is presented in Table 5. Uncertainty was calculated for the total dataset as well as the four subsets (splitting the total dataset a function of PM_2.5_ levels and seasons). 8-h averaged values were used for this analysis. As reported in Table 5, the uncertainty was higher than 2.5 µg/m^3^ in the case of the total database and for winter and high-concentration (i.e., >18 µg/m^3^) datasets, thus, indicating unsuitable performances of one or both the co-located instruments. Contrariwise, the uncertainty was lower than 2.5 µg/m^3^ when considering the summer and low-concentration (i.e., <18 µg/m^3^) datasets, thus, indicating better performance under these conditions. Therefore, this analysis outlined the potential presence of seasonal and proportional biases that must be verified.

For simplicity and considering the substantial level of agreement as outlined in the previous evaluations, all further statistical analyses were carried out with the variable ABx, i.e., the mean of the data for the three co-located ABs. Analysis for each AB is reported in the supplementary material. 

### 3.3. Accuracy: Comparison with Reference Methods

Despite the low number of sampling sessions, the non-parametric Mann-Whitney test was performed as the first analysis to assess the differences between two independent groups of a continuous variable. A non-parametric test was chosen as it was verified that the AB concentration data (as well as in the case of Aerocet and OPC) were not normally distributed (Kolmogrov-Smirnov test).

In this study, the concentration data obtained from all direct-reading instruments in each session were averaged on an 8-h basis and compared with the gravimetric PM_2.5_ concentrations. As reported in Table S2, the obtained results clearly show statistically non-significant differences between the median concentrations of all direct-reading devices and the gravimetric method.

Table 6 (and Table S3) shows the correlation coefficients between the direct-reading monitors (ABx, Aerocet, and OPC–8-h averaged data) and the gravimetric method EPA WINS. The results revealed high correlation values between ABx and the gravimetric methods (rho = 0.916) and between ABx and the other direct-reading instruments (rho = 0.991 and 0.932 for Aerocet and OPC, respectively) (Table 6).

Correlations between direct-reading instruments were also performed on 1-min averaged data (Table 7 and Table S4), and, as expected, ABx was found to be highly correlated with the other direct-reading devices (ABx vs. Aerocet: 0.982 (rho); ABx vs. OPC: 0.987 (rho)).

To assess the level of agreement between direct-reading instruments and the gravimetric method, a linear regression analysis was performed on the entire dataset, while considering ABx, Aerocet, and OPC concentrations as the dependent variable (y) and the reference gravimetric method concentrations as the independent variable (x). Table 8 reports the regression parameters between ABx, Aerocet, and OPC (averaged on 8-h basis) and the gravimetric method EPA WINS. Results concerning each AB are shown in the Supplementary Material (Table S5 and Figure S4).

As reported in Table 8, the highest R^2^ value was reached between ABx and EPA WINS (R^2^: 0.826), while R^2^ for Aerocet and OPC were slightly lower (0.808 and 0.769, respectively). Additionally, to evaluate the comparability between the two methods, the indications that were summarized by Watson et al. [42] were followed. Evaluating these criteria, it is clear that Aerocet and OCP could not be considered mutually predictable and comparable with respect to the reference method, because slope and intercept criteria were not met and R values were always <0.9. Contrariwise, ABx can be considered as comparable but not mutually predictable with respect to EPA WINS because R met the criteria reported above (which does not occur for slope and intercept parameters). The regression parameters between the direct-reading methods are reported in Table 9 and Table S6.

Despite the reduced sample size (9–10 samples per season), the linear regression analysis was also performed separately during summer and winter to evaluate the concordance between the direct-reading monitors and the gravimetric method across different climatic conditions and PM_2.5_ concentrations. The results (Table 10 and Table S7) indicate that during summer and at lower concentrations, R^2^ for all comparison analyses were higher than the R^2^ outcomes for winter comparisons, thus, confirming the indication of a better performance under these conditions, as outlined by the uncertainty analysis (Table 5).

### 3.4. Accuracy: Measurement Error Trends

To better evaluate the possible errors and error trends, instruments were also analyzed by using the Bland-Altman plot method [45,46]. The single plots for each AB are reported in Figure S5. The results revealed good agreement between the two techniques, especially for lower concentrations (i.e., <20 µg/m^3^); however, they also showed an error that tended to increase with increasing PM_2.5_ concentrations.

**Figure 1:**
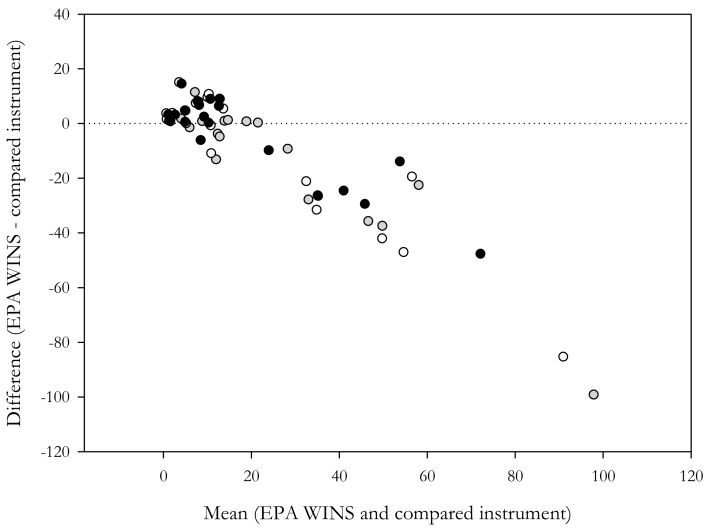
Bland-Altman plot for different instruments (grey: Aerocet; white: Optical Particle Counters (OPC); black: ABx). The mean concentrations between the gravimetric reference method (EPA WINS) and the compared instrument are reported on the x-axis while on the y-axis the differences between methods are shown (8-h average). The dotted line represents the perfect agreement between the two instruments (absolute deviation: 0).

Therefore, to evaluate whether the error increase was influenced by an increase in PM concentrations and not by an instrument drift over time, the Bland-Altman plot analysis was carried out while considering the differences between all direct-reading instruments (Figure 1). The Bland-Altman plot (Figure 1) clearly shows that all the direct-reading instruments were characterized by the same trend (increase in the absolute error with increase in PM_2.5_ concentrations).

Regarding the relative error analysis between direct-reading instruments and the gravimetric method, as reported in Table 11, the ABx relative error for summer was very similar to the summer OPC relative error, but five times higher than relative error that was calculated between Aerocet and EPA WINS. Contrariwise, during winter, the average relative error calculated for AB was equal to half the relative error calculated for the other methods (OPC and Aerocet). When considering each single monitoring session (Table S8), the ABx relative error was lower than the OPC relative error in 66.6% of the cases and lower than the Aerocet relative error in 52.6% of the cases.

Similar results were obtained with the absolute error analysis (Table 11). Additionally, the absolute error for ABx during summer differed by less than 1 µg/m^3^ from the OPC absolute error but was five times higher than the Aerocet error. During winter, the average AB absolute error was equal to half of the absolute errors for OPC and Aerocet. While considering each single session (Table S9), the ABx absolute error was lower than the OPC error in 68.4% of the cases and lower than Aerocet absolute error in 52.6% of the cases.

Relative and absolute errors (Table 11) were negative during summer and positive during the winter sessions, indicating an underestimation and overestimation of concentration data during summer and winter, respectively.

**Figure 2:**
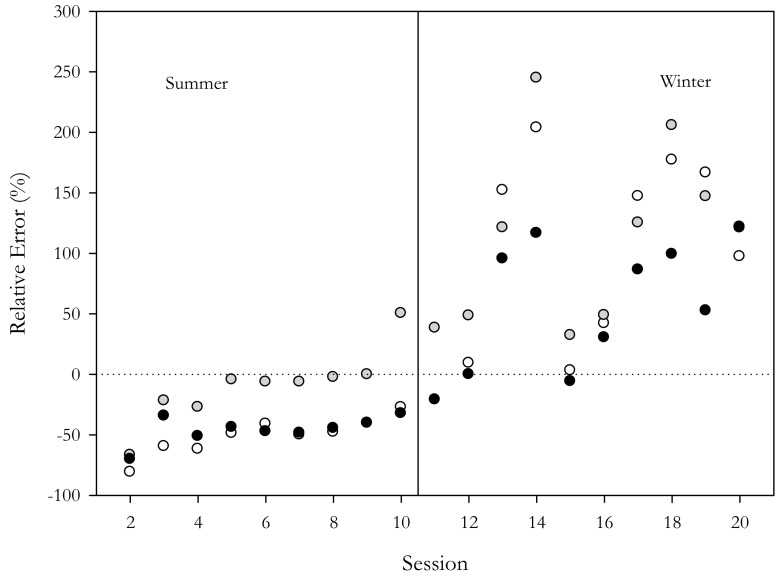
Relative error trend. The abscissa axis x reports the number of monitoring sessions while the ordinate axis y shows the relative error for the different instruments (grey: Aerocet; white: OPC; black: mean of ABx) as compared to the gold standard (gravimetric method).

To evaluate the relative error trend and to assess the relationship between the AB error and instrument drifts, the relative errors of all the selected direct-reading instruments were plotted vs. time (Figure 2). Figure 2 reports the ascending order of the monitoring sessions on the abscissa x and the relative error (%) between the direct-reading instrument and the gravimetric methods on the ordinate. The figure clearly indicates that summer data are characterized by a lower relative error and lower instrumental differences than the winter data. Further, the error trend was similar for all of the tested instruments, suggesting the lack of instrument calibration drifts.

Finally, when considering the seasonal averaged ratio between the direct-reading instruments and the gravimetric method, different correction factors have been proposed for ABs, Aerocet, and OPC. In particular, the summer correction factors (calculated as the ratio between the reference PM concentrations and those measured by direct-reading instruments [20]) for ABs, Aerocet, and OPC are 0.54, 0.90, and 0.49 for summer and 1.58, 2.13, and 2.11 for winter, respectively. 

### 3.5. Error and Meteorological Parameters

Finally, to evaluate whether meteorological parameters could affect the performances of ABs and other devices, a correlation analysis between errors (both absolute and relative errors) and meteorological variables (temperature, atmospheric pressure, wind intensity and direction) was performed. Rainfall has not been considered because it was absent during the entire monitoring period. As reported in Table 12, absolute errors between ABx (and also between the other direct-reading methods) and the gravimetric method were positively and highly correlated with RH and wind intensity and negatively correlated with wind direction. A moderate and negative correlation was also found with temperature. Contrariwise, the relative error was, in general, less correlated than the absolute error with the same meteorological parameters.

Moreover, despite the low number of acquired samples and variables, a multiple linear regression analysis was performed between ABx absolute error (compared with the gravimetric method) and meteorological parameters that were measured at the sampling point (Table 13). In the model, the absolute error was included as the dependent variable and meteorological parameters (temperature, RH, atmospheric pressure, wind intensity, and wind direction) as predictors. Only meteorological variables that were found to be statistically significant in the bivariate correlation analysis (at a *p*-value <0.05) were considered in the multiple regression model. The results from this analysis must be carefully evaluated, mainly due to the low sample number and variables considered (N = 19). However, preliminary results, as reported in Table 13, indicate that RH exhibited the main influence on ABx absolute error.

## 4. Discussion

In this study, PM_2.5_ MMs were tested at an urban background station to evaluate their performance against the reference gravimetric method for PM_2.5_ (EPA WINS) and other common and widely used portable direct-reading instruments (Aerocet and OPC).

First, the tested ABs were mutually compared by linear regression analyses between the co-located instruments (Table 4). As reported in other studies, results in this study showed good precision among ABs throughout the entire monitoring period [31]. In particular, different AB copies can be classified as comparable to each other, even if not being characterized by mutual predictability. ABs were also comparable but not mutually predictable when compared to other traditionally used portable PM monitors (Aerocet). The uncertainty between couples of ABs was moderate during the entire study period (Table 5), even if not fully compliant with the uncertainty criterion proposed by the EC working group [43] (i.e., uncertainty <2.5 µg/m^3^). Overall, these results show that ABs are characterized by good precision; however, some factors can interfere in defining measurement error that can potentially affect the precision and accuracy of the results (i.e., RH and PM_2.5_ concentration).

It was observed that MMs tended to overestimate EPA WINS concentrations during winter and underestimate the reference concentrations during summer (Table 2, Figure 2). The regression analysis performed on the total dataset (Table 8) showed a regression slope significantly different from 1 with good R^2^ values, indicating the presence of a proportional bias. Such bias could be related to differences in the PM that were monitored at the sampling point with respect to the standard particulate used for instrument calibration [21]. It is well known that the factory calibration factor of a photometer cannot be used to obtain accurate data when there are marked differences in terms of shape, morphologies, size-distribution, chemical composition, and reflectance properties between the analyzed particulate and the standard dust. As reported in different studies that were conducted in the study area [20,36,48], the local urban particulate is typically less dense than the standard dust, which could result in a significant overestimation of PM concentrations by optical particle counter and nephelometers. This can explain the underestimation of average concentrations by a factor of about 0.5 in summer and an overestimation of mean concentrations by a factor of three during winter. These results are in accordance with those reported by Mukherjee [31] which showed that AB tended to underestimate or overestimate PM_2.5_ concentrations depending on the aerodynamic diameters of the particles. Indeed, it was shown that, with larger particles, AB seemed to underestimate PM_2.5_ concentrations whereas when the smallest fraction was predominant, PM concentrations tended to be overestimated. This is the case for the winter size-distribution at the sampling site, which is characterized by a sharp increase in the accumulation-mode peak during the cold season [36].

Further, it should be noted that all the instruments used in the field campaign (AB, OPC, and Aerocet) showed the same error trend over time (Figure 1 and Figure 2) and were characterized by a high overestimation error during winter and a slight underestimation error during summer when PM_2.5_ concentrations were lower. Thus, it is reasonable to exclude the presence of an instruments drift over time and to assume the presence of a seasonal bias. 

The regression analysis between EPA WINS and the mean of AB concentrations showed a high R^2^ value (R^2^ > 0.80), which is in agreement with the R^2^ value calculated by manufacturers for regression between ABs and the gravimetric method and used as reference method [49] (R^2^ = 0.70). However, as expected, ABs (like other instruments tested in this study (Aerocet and OPC) cannot be classified as mutually predictable with respect to the gravimetric method in the concentration range under investigation (2.3–48.3 µg/m^3^). However, ABx (considered as the average of ABs) can be considered to be comparable to the gravimetric method (unlike the other direct-reading instruments tested). 

Also, the Bland-Altman plot analysis showed a negative error trend that increased with increasing PM_2.5_ concentrations (especially at concentrations >25 µg/m^3^) for all instruments (Figure 1). The value of 25 µg/m^3^ can be considered as a threshold above which the performance of instruments significantly decreases in accordance with the results that were reported by the manufacturers [49] and elsewhere [34]. However, it should be noted that Johnson et al. [34]. evaluated the same sensor that was used in the ABs and indicated a suitability for PM concentrations <50 µg/m^3^. Therefore, while the level of 25 µg/m^3^ cannot be used as a clear demarcation value in terms of sensor performance, it should be remembered that the average annual concentrations of PM_2.5_ across Europe are usually lower than this threshold [50] and can be overcome in particular microenvironments [51,52,53], especially during short-term periods [48].

The error associated with direct-reading methods could be reduced by using appropriate calibration factors. As reported in several studies, calibration factors can be calculated as the ratio between the reference PM concentrations and those that were measured by direct-reading instruments [20]. In this study, calibration factors were calculated separately for the two monitoring seasons (and as a function of PM_2.5_ concentration), since the performance of ABs varied significantly with season. Once corrected on the basis of EPA WINS PM_2.5_ concentrations, AB performances were significantly improved (R^2^ for comparison: AB1 vs. EPA WINS: 0.82; AB2 vs. EPA WINS: 0.82; AB3 vs. EPA WINS: 0.83) and all the ABs could be considered comparable to the gravimetric method. Therefore, correction factors should be used to obtain reliable concentrations by direct-reading instruments. As reported by Mukherjee et al. [31], the bias between ABs and the comparison instruments depended on the size distribution and chemical composition of the aerosol. It is important to note that the response of optical-based sensors is a function of aerosol properties at the specific sampling point (such as size distribution and chemical composition) [34] and the relationship between light scattered by the instrument and PM concentrations is set a priori by manufacturers using well characterized standard dust. The challenge with optical measurement techniques arises when the instruments measure PM that differs from the PM used for instrument calibration [21]. In this study, the correction factor was calculated for every comparison session and reported as a summer/winter mean correction factor. However, it is important to state that it must be calculated in a specific way (depending on the sampling period and location) for different monitoring sessions, and, for this reason, it should not be used in other contexts. Furthermore, in the case the correction factor is not calculated and not taken into account, it should be considered that the introduced error may not be negligible (as in the case of direct-reading error reported in this study).

The influence of RH on the instrument performance and, in particular, on the light scattering methods, has already been analyzed in previous studies [20,54]. According to these investigations, a moderate high correlation (mean between AB: 0.589) between the (absolute) measurement error and RH was found and confirmed by multivariate analysis (Table 13). The results from the multivariate analysis confirmed the findings of the univariate analyses, namely, a significant relationship between absolute ABx error and RH, which was found to explain about 46% of the total variability in the multivariate model.

Some studies have reported the influence of RH on different particle properties, such as: (i) particle volume; (ii) shape; (iii) refractive index; and, consequently, (iv) light scattering properties [54,55]. Additionally, the AB manufacturer [49] indicates that the RH (>80%) has a negative effect on the accuracy of instrumental responses because aerosols take on water and become more reflective at high RH conditions. As reported in Figure S6–S10, the effects of RH on absolute and relative errors also seem to occur at lower RH values than those that were proposed by the manufacturer, especially in the presence of high PM concentrations (i.e., >25 µg/m^3^). Lower errors seem to occur at RH values below 50% even when the PM concentrations are generally lower. Effects of RH on performance of low-cost PM sensors are reported in a recent study [56], and the results indicate that RH may also cause condensation on electrical components, leading to a resistive bridge across components. As reported above, the performance of AB was worst during winter when the average RH measured at the sampling point was 71.5% and better during the summer session which was characterized by lower RH (40.7% on average). The combined effect of RH and PM concentrations as a factor that focuses on the measurement error should be further explored in future studies to expand on case studies of data measured in the field under different conditions.

This study was specifically conducted during two different seasons that were characterized by different environmental conditions (PM concentrations, temperature, RH, etc.) to evaluate instrument performance across several conditions. The error trend that was reported in this study could not be reasonably related to a single environmental factor but was related to the total contribution by different conditions, such as the increase in PM concentrations and an increase in RH. Therefore, it would be useful to perform laboratory tests in future studies to evaluate the effects of single potential error determinants on the error trend.

### 4.1. Practicality

The present study was conducted with the primary aim of evaluating the performance of ABs and their potential applicability in exposure assessment studies. It should be noted that despite these devices not being intended for use in techniques equivalent to gravimetric methods, these devices were compared to a reference filter-based method and to other direct-reading instruments that are widely used in the scientific literature and already evaluated elsewhere. For example, Spinazzè et al. [20] recently assessed the performance of different direct-reading methods (Aerocet and OPC) and gravimetric instruments at the same sampling point used in our study. As reported by Spinazzè et al. [20], portable direct-reading methods are easy to operate and are able to provide data at high temporal resolutions. Contrariwise, filter-based methods are generally not able to provide information at high spatial and temporal resolutions, which is an essential feature for monitoring environments characterized by high variability in terms of pollutant concentrations, such as urban environments [10]. The AB device tested in our investigation is smaller than the other widely used direct-reading instruments commercially available, cheaper than the other investigated devices, easy to transport and user-friendly, and able to provide additional data on temperature and RH. Moreover, it is associated with an Android application that affords an instant view of the concentration data and a facility of data-interpretation, even to the general population. Moreover, it is also possible to detect PM_2.5_ maps and graphs in real-time directly from the smartphone. Finally, despite the fact that the AB cannot be considered to be mutually predictable, but only comparable with respect to the reference method and that its performance seems to be influenced by different variables (RH and PM concentrations), we found a similar performance trend across different direct-reading instruments, such as Aerocet and OPCs, already widely used in human exposure assessment studies [53,56,57,58,59,60,61,62,63].

In addition to poor agreement with the reference method, another disadvantage that is related to the use of ABs is due to the data communication protocol. As mentioned above, data acquired by AB are sent to an Android application via Bluetooth and then stored. As can be seen in Table 2, the monitoring time (reported as the number of data points used during statistical analysis) is different for the three ABs because during the monitoring session the Bluetooth connection between AB and mobile phone could be lost.

To summarize, despite the disadvantages that are reported above and mainly related to the presence of a measure bias and to connection loss, AB could be used, with some precaution (i.e., application of a proper correction factor, management of potential outliers in the data series), across different and several applications. As reported by other authors [31], such sensors can be useful to assess the short-term changes in aerosol environment due to their acquisition rate and high response. Moreover, like other MMs, AB can potentially: (i) provide real-time data at high spatial and temporal resolutions; (ii) collect data across long or short-term campaigns and as stationary or mobile devices; (iii) collect data across different environments, both indoors and outdoors; (iv) be used for the evaluation of PM hot-spots; (v) be used as a support to fixed air quality monitoring stations; (vi) collect data at personal or individual levels, thus, enabling the subject to carry out the measurement themselves; and, (vii) provide pollutant data regarding community/individual exposure, or regarding a selected category of subjects (such as workers or susceptible subjects) [28,33]. 

Regarding the potential use in human exposure assessment studies, AB and MMs, in general, potentially have the ability to improve knowledge and become a novel way for human exposure assessment due to the advantages reported above, low costs, and their ability to measure pollutants across different environments, scenarios, and applications. One such application concerns the new paradigm of “citizen science” (the pros and cons of which should be carefully evaluated) [28] being applied by the AirBeam—Aircasting application (http://aircasting.org).

### 4.2. Strengths and Limitations of The Study

The main limitation of this study is related to the low number of sampling sessions (N = 20) over the monitoring period, which are further reduced if the two different monitoring sub-periods (warm and cold period), specifically identified to evaluate the performance of MMs across different climatic conditions and at different PM_2.5_ concentration levels, are considered.

Additionally, the portable instruments were evaluated only at a fixed site station and not under their normal use conditions, namely, as personal devices. A further development of this study will include the evaluation of AB performance as compared to other portable monitors for personal exposure measurement applications. Moreover, the monitoring sessions were carried out only at one urban background site, not allowing the assessment of possible spatial variations in the monitoring area. Further, despite reference methods and accepted standard practices were adopted for gravimetric sampling, the adoption of further precautions, and technical measures (i.e., field blanks, duplicated measurements, etc.) would have allowed for further control and reduction of the level of variability of the PM_2.5_ gravimetric measurements. Finally, the changes in the AB performances were assessed only within a relatively restricted concentration range (2.3–48.3 µg/m^3^), even though this is typical of a medium-sized provincial town, such as Como. In this context, the authors think that evaluations conducted at higher PM concentrations could be relevant because, as reported by the manufacturer [49], the relation between AB and the reference methods should become increasingly non-linear above 100 µg/m^3^ [34]. Despite the results of different studies for PM sensors are quite difficult to compare among each other (as the responses of these sensors may be influenced by aerosol composition), it should be noted that a recent study that was performed in the framework of AQ-SPEC project by Feinberg et al., 2018 [64], concerning the long-term evaluation of air sensors, outlined that AB is one of the sensors with the highest correlation with reference measurements, despite they may have a certain level of measurement noise and a potential level of interference related to the presence of relative humidity. Anyhow, further studies for PM miniaturized sensors are needed to in deep evaluate their performance for different air pollutant concentration ranges and aerosol characteristics, both in (i) long-term, in-field studies [64] and under controlled conditions [65]. 

Therefore, additional studies covering a wider range of PM_2.5_ concentrations and assessing further influencing factors (e.g., particles size and shape, particles refractive index, etc.) on measurement errors are suggested and encouraged.

Despite the aforementioned limitations, one of the main advantages of the present study is that, to the knowledge of the authors, this is one of the first comparison studies on ABs conducted in real environmental conditions and not only through laboratory tests. The possibility to quantify the instrument performances under real-world conditions is indeed a key highlight of this study [31] because, in general, laboratory tests can hardly reproduce an aerosol mixture matching the complex composition and variability of particles in real environments [34]. However, field tests can provide a greater variation of conditions in contrast to the controlled conditions that were found in laboratory tests [66].

## 5. Conclusions

In conclusion, despite a moderate level of agreement between AB and the gravimetric method, especially at lower concentrations, relevant bias was found across the entire sampling period, indicating the necessity to develop standardized protocols and harmonize performance evaluation criteria for these devices. Moreover, it is important to interpret data outcomes from AB (and, in general, from optical particle counters and photometers) carefully, especially if appropriate calibration factors are not used. However, that very similar trends in performances were found to those of other widely used direct-reading instruments (Aerocet and OPC), should be underlined; although, all instruments that were compared are based on the same measurement technique.

Future developments should aim at evaluation of AB, and, in general, of MMs, across different environments that are characterized by different PM concentrations and chemical-physical characteristics. Furthermore, the influences of meteorological and other environmental parameters should be better evaluated. Also, AB should be evaluated over a longer time-period and under the same conditions in which the instruments are actually used: as personal and mobile monitors. Evaluation of measurement instruments in real-word conditions and during real operation procedures can provide more information regarding the performance of instruments and their usability. In this regard, other tests should be performed under real use conditions to evaluate the response of subjects to the use of the instrument itself (in terms of portability, ease of use, interference with normal activities, etc.).

## Figures and Tables

**Table 1:** Principal outcomes from other studies that evaluated AirBeam (AB). n.a.: not available.

Reference	MonitoRing Period	Sampling Point	Compared Instruments	Performed Analysis	Notes
[31]	12 weeks	Cuyama Valley (California, USA). Field test	GRIMM 11-R Met One (BAM)	Precision Accuracy Evaluation of sampling orientation Size distribution Meteorology and size distribution influence	High precision between couple of ABs: R^2^ > 0.95 Low R^2^ for comparison between AB and BAM (<0.33) Instruments were evaluated over different meteorological conditions and aerosol properties Authors used the default conversion algorithm that was used to convert counts to PM concentrations (PM_2.5_: 0.518 + 0.00274 × particle count − hppcf)
[32]	n.a	Laboratory test	Personal DataRAM 1500, Thermo Scientific, Waltham, MA, USA	Tests performed across different occupational settings Regression analysis Bias analysis Precision analysis	R^2^ from comparison with comparison instrument: 0.7–0.96 High precision: 2–9% Precision < 10% for all types of aerosol used (salt, welding fume, ARD) AB is not able to detect mass concentrations > 200 µg/m^3^
[33]	2013–2014	USA. Field test	Met One (BAM) FEM	Regression analysis OLS regression	R^2^ ranges from 0.65 and 0.66

**Table 2:** PM_2.5_ concentrations acquired with different monitoring devices. N: number of data point used for statistical analysis; Min: minimum; Max: maximum; S.D.: standard deviation.

Data averaged for 1-min	**PM_2.5_—Total Summer Dataset (µg/m^3^)**						
		**N**	**Mean**	**Median**	**Min.**	**Max.**	**S.D.**
	AB 1	3816	7.1	7.2	0.7	17.8	4.7
	AB 2	3862	6.5	6.1	0.7	15.6	4.2
	AB 3	3259	6.8	6.4	0.6	18.0	4.8
	Aerocet	4544	12.3	11.5	0.3	33.9	8.9
	OPC	4530	6.6	6.2	0.2	17.3	4.7
	**PM_2.5_—Total Winter Dataset (µg/m^3^)**						
		**N**	**Mean**	**Median**	**Min.**	**Max.**	**S.D.**
	AB 1	3782	34.9	33.3	0.8	104.1	29.5
	AB 2	3574	40.8	38.7	0.9	108.9	32.3
	AB 3	3833	37.9	42.5	0.7	95.0	28.5
	Aerocet	4645	50.8	43.7	0.8	202.6	46.5
	OPC	4097	52	40.9	0.8	313.2	50.1
8-h data	**PM_2.5_—Total Summer Dataset (µg/m^3^)**						
		**N**	**Mean**	**Median**	**Min.**	**Max.**	**S.D.**
	AB 1	9	7.0	8.0	1.3	13.2	4.7
	AB 2	9	7.2	8.0	1.3	13.0	4.6
	AB 3	9	7.0	8.0	1.2	13.3	4.7
	Aerocet	9	12.7	14.1	1.4	28.5	9.4
	OPC	9	6.8	7.6	0.8	13.8	4.9
	EPA WINS	9	12.5	14.8	2.3	21.7	7.2
	**PM_2.5_—Total Winter Dataset (µg/m^3^)**						
		**N**	**Mean**	**Median**	**Min.**	**Max.**	**S.D.**
	AB 1	10	38.1	39.8	5.3	98.1	31.0
	AB 2	10	41.4	40.3	4.7	102.7	33.2
	AB 3	10	36.1	36.1	5.0	88.0	27.1
	Aerocet	10	50.3	47.9	7.0	147.8	41.9
	OPC	10	47.8	47.1	0.0	133.9	41.7
	EPA WINS	10	22.8	20.5	5.3	48.3	15.8

**Table 3:** Meteorological parameters at the sampling site during the two monitoring periods. N: number of data used in statistical analysis; Min: minimum; Max: maximum; S.D.: standard deviation

Meteorological Data—Total Summer Dataset					
	Mean	Median	Min.	Max.	S.D.
ARPA cumulative rainfall (mm)	0.0	0.0	0.0	0.2	0.0
Temperature (°C)	29.2	29.8	17.1	38.7	5.1
RH (%)	40.7	34.9	16.1	82.6	17.1
Atmospheric pressure (hPa)	1002.6	1002.5	993.9	1009.0	5.0
Wind intensity (m/s)	0.9	0.9	0.1	1.7	0.3
Wind direction (°)	186.4	198.0	2.0	267.0	64.3
**Meteorological Data—Total Winter Dataset**					
	Mean	Median	Min.	Max.	S.D.
ARPA cumulative rainfall (mm)	0.0	0.0	0.0	0.0	0.0
Temperature (°C)	8.0	8.8	−0.9	14.0	3.2
RH (%)	67.8	72.4	23.9	99.9	21.1
Atmospheric pressure (hPa)	1005.4	1003.4	992.6	1022.3	8.6
Wind intensity (m/s)	39.5	0.5	0.0	229.0	75.4
Wind direction (°)	145.1	173.0	0.0	249.0	88.5

**Table 4:** Regression parameters between AB (data averaged on a 1-min basis). N: number of data; R: Pearson correlation coefficient; *p*: significance; m: slope; q: intercept; SE: standard error. Regression parameters that did not meet the Watson et al. criteria are marked in bold while values that met these criteria are underlined.

Instrument Compared	Regression Model				Slope			Intercept		
	N	R	R^2^	*P*	m	SE	*p*	q	SE	*p*
AB1 vs. AB2	6188	0.995	0.990	<0.001	**0.978**	0.001	<0.001	**0.018**	0.001	<0.001
AB1 vs. AB3	5862	0.994	0.988	<0.001	**1.004**	0.001	<0.001	0.004	0.002	0.037
AB2 vs. AB3	5761	0.995	0.990	<0.001	**1.027**	0.001	<0.001	**−0.011**	0.002	<0.001
AB1 vs. AB2	Comparable and mutually predictable: NO Comparable but not mutually predictable: YES									
AB1 vs. AB3										
AB2 vs. AB3										

**Table 5:** Results of uncertainty analysis conducted between couple of co-located instruments. High-concentration database refers to particulate matter_2.5_ (PM_2.5_) concentrations ≥18 µg/m^3^ while the low-concentration database refers to PM_2.5_ concentrations <18 µg/m^3^. N: number of sessions considered in the analysis. In bold and underline are marked results that are not in agreement with the criterion followed in this test (>2.5 µg/m^3^).

	AB1-AB2 (µg/m^3^)	AB1-AB3 (µg/m^3^)	AB2-AB3 (µg/m^3^)
Total database (N: 20)	**2.58**	**2.80**	**4.25**
High concentration (>18 µg/m^3^) (N: 6)	**4.02**	**4.39**	**7.71**
Low concentration (<18 µg/m^3^) (N: 14)	1.60	1.72	0.60
Summer (N: 10)	0.32	0.27	0.29
Winter (N: 10)	**3.63**	**3.95**	**6.01**

**Table 6:** Correlations between all instruments (8-h averaged data). All the correlations are significant at 0.001 level and results are based on 19 monitoring sessions. Spearman’s rank order correlation (rho) is reported in the table.

	ABx	Aerocet	OPC	EPA WINS
ABx	---	0.991	0.932	0.916
Aerocet	---	---	0.940	0.932
OPC	---	---	---	0.821
EPA WINS	---	---	---	---

**Table 7:** Correlations between direct-reading instruments (1-min average). All correlations are significant at 0.001 level. Spearman’s rank order correlation (rho) is reported in the table.

	ABx	Aerocet	OPC
ABx	---	0.982 (N = 9009)	0.987 (N = 8467)
Aerocet	---	---	0.989 (N = 8429)
OPC	---	---	---

**Table 8:** Regression parameters between direct-reading instruments (8-h averaged data) and the gravimetric method. N: number of data; R: Pearson correlation coefficient; *p*: significance; m: slope; q: intercept; SE: standard error.

Instrument Compared	Regression Model				Slope			Intercept		
	N	R	R^2^	*p*	m	SE	*p*	q	SE	*p*
ABx vs. EPA WINS	9	0.909	0.826	<0.001	1.849	0.206	<0.001	−9.522	4.543	0.051
Aerocet vs. EPA WINS	9	0.899	0.808	<0.001	2.428	0.287	<0.001	−11.042	6.336	0.099
OPC vs. EPA WINS	9	0.877	0.769	<0.001	2.397	0.319	<0.001	−14.593	7.059	0.054
	**Comparable and Mutually Predictable**					**Comparable But Not Mutually Predictable**				
ABx vs. EPA WINS	NO					YES				
Aerocet vs. EPA WINS	NO					NO				
OPC vs. EPA WINS	NO					NO				

**Table 9:** Regression parameters between direct-reading instruments (1-min averaged data). N: number of data; R: Pearson correlation coefficient; *p*: significance; m: slope; q: intercept; SE: standard error.

Instrument Compared	Regression Model			Slope			Intercept		
	R	R^2^	*p*	m	SE	*p*	q	SE	*p*
Abx vs. Aerocet	0.928	0.861	<0.001	0.644	0.003	<0.001	2.167	0.134	<0.001
Abx vs. OPC	0.876	0.767	<0.001	0.575	0.003	<0.001	6.632	0.170	<0.001
	**Comparable and Mutually Predictable**					**Comparable But Not Mutually Predictable**			
ABx vs. Aerocet	NO					YES			
ABx vs. OPC	NO					NO			

**Table 10:** Regression parameters between direct-reading instruments and EPA WINS (8-h averaged data). Regression parameters were calculated and reported for the summer and winter datasets. N: number of data; R: Pearson correlation coefficient; *p*: significance; m: slope; q: intercept; SE: standard error.

Summer Database										
Instrument Compared	Regression Model				Slope			Intercept		
	N	R	R^2^	*p*	m	SE	*p*	q	SE	*p*
ABx vs. EPA WINS	9	0.984	0.968	<0.001	0.629	0.043	<0.001	−0.801	0.619	0.237
Aerocet vs. EPA WINS	9	0.940	0.884	<0.001	1.222	0.168	<0.001	−2.582	2.395	0.317
OPC vs. EPA WINS	9	0.969	0.939	<0.001	0.660	0.063	<0.001	−1.429	0.905	0.159
**Winter Database**										
**Instrument Compared**	**Regression Model**				**Slope**			**Intercept**		
	**N**	**R**	**R^2^**	***p***	**m**	**SE**	***p***	**q**	**SE**	***p***
Abx vs. EPA WINS	10	0.943	0.889	<0.001	1.808	0.225	<0.001	−2.670	6.129	0.675
Aerocet vs. EPA WINS	10	0.901	0.812	<0.001	2.380	0.406	<0.001	−3.975	11.094	0.729
OPC vs. EPA WINS	10	0.900	0.810	<0.001	2.369	0.405	<0.001	−6.212	11.059	0.590

**Table 11:** Relative and absolute errors (mean ± S.D.; median, minimum, maximum) calculated between direct-reading instruments and the gravimetric method. The error is reported considering the mean values during summer and winter monitoring periods as well as the entire dataset.

		ABx		Aerocet		OPC	
		Mean (±S.D.)	Median (Min.; Max.)	Mean (±S.D.)	Median (Min.; Max.)	Mean (±S.D.)	Median (Min.; Max.)
Relative Error (%)	Total database	9	−27	55	38	23	−27
		(±64)	(−70; 122)	(±82)	(−67; 245)	(±98)	(−100; 204)
	Summer database	−46	−45	−10	−6	−51	−49
		(±10)	(−70; −32)	(±29)	(−67; 50)	(±14)	(−81; −27)
	Winter database	58	86	113	121	90	122
		(±51)	(−21; 122)	(±69)	(32; 245)	(±93)	(−100; 204)
Absolute error (µg/m^3^)	Total database	5.7	−0.8	14.6	4.0	10.5	−1.3
		(±15.5)	(−8.8; 47.9)	(±24.0)	(−2.9; 99.4)	(±24.8)	(−10.5; 85.6)
	Summer database	−5.5	−6.1	0.2	−0.6	−5.7	−5.2
		(±2.7)	(−8.8; −0.8)	(±3.4)	(−2.8; 9.5)	(±2.6)	(−9.3; −1.3)
	Winter database	15.7	12.1	27.5	24.7	25.0	20.5
		(±15.4)	(−2.2; 47.9)	(±27.0)	(1.7; 99.4)	(±26.8)	(−10.5; 85.6)

**Table 12:** Correlations between absolute and relative errors (direct-reading instruments vs. EPA WINS) and meteorological parameters (RH: relative humidity; Atm. pressure: atmospheric pressure; Wind int.: wind intensity; Wind dir.: wind direction). ** Correlation is significant at the 0.01 level (2-tailed); * Correlation is significant at the 0.05 level (2-tailed).

		Temperature (°C)	RH (%)	Atm.Pressure (hPa)	Wind Int. (m/s)	Wind Dir. (°)
Absolute error						
ABx	Pearson correlation	−0.495 *	0.690 **	0.317	0.749 **	−0.788 **
Aerocet	Pearson correlation	−0.584 *	0.685 **	0.314	0.726 **	−0.778 **
OPC	Pearson correlation	−0.568 *	0.734 **	0.353	0.775 **	−0.807 **
Relative error						
ABx	Pearson correlation	−0.400	0.339	-0.231	0.431	−0.453
Aerocet	Pearson correlation	−0.710 **	0.488 *	-0.113	0.436	−0.492 *
OPC	Pearson correlation	−0.639 **	0.541 *	-0.115	0.477 *	−0.471 *

**Table 13:** Summary of the multiple regression model results. Both unstandardized (B) and standardized (Beta) coefficients and the standard error (SE) for each independent variable, the model statistical significance (Sig.), and the upper and lower 95% confidence intervals (95% C.I.) for beta are reported. Other parameters are reported as indicators of the regression model: R, R^2^, adjusted R^2^ (Adj. R^2^), standard error (Std. Error), and p value (*p*). * Variable is significant at the 0.05 level (2-tailed).

ABx (µg/m^3^)						
Independent Variable (Predictors)	Unstandardized Coefficient		Standardized Coefficient	Sig.	95% C.I.	
	B	SE	Beta		Lower	Upper
(Constant)	4.406	19.285		0.823	−37.256	46.069
Temperature (°C)	0.052	0.225	0.046	0.820	−0.433	0.538
RH (%)	0.312	0.136	0.469 *	0.039	0.018	0.607
Wind intensity (m/s)	0.056	0.075	0.255	0.466	−0.105	0.217
Wind direction (°)	−0.070	0.067	−0.365	0.311	−0.215	0.074
**Regression Model Statistics**						
R	R^2^	Adj. R^2^	Std. Error	*p*		
0.883	0.780	0.712	6.77141	<0.001		

## Supplementary Materials

The following are available online at http://www.mdpi.com/1424-8220/18/9/3089/s1, Figure S1: Setup of the sampling equipment and relative position (view from the above). Figure S2: Wind direction (°) and intensity (m/s) during warm and cold periods. Figure S3: Maps of wind direction and intensity at the sampling point during cold and warm periods. Red areas correspond to wind intensity ≥1.50 m/s, yellow areas to wind intensity between 1 and 1.5 m/s, green areas between 0.5 and 1 m/s, and blue to wind intensity between 0 and 0.5 m/s. Table S1: Error between ABs - descriptive statistic. S.D.: standard deviation; Max.: maximum; Min.: minimum; C.I.: confidence interval. Table S2: Mann-Whitney test statistics. Z: Mann-Whitney test statistics; Asymp. Sig: significance. Table S3: Correlations between all ABs (8-h averaged data). All correlations are significant at 0.001 level and results are based on 19 monitoring sessions. In the table is reported the Spearman’s rank order correlation (rho). Table S4: Correlations between direct-reading instruments (1-min averaged data). All correlations are significant at 0.001 level. In brackets are reported the number of data used for analysis. In the table is reported the Spearman’s rank order correlation (rho). Table S5: Regression parameters between direct-reading instruments (8-h average) and the gravimetric method. N: number of data; R: Pearson correlation coefficient; p: significance; m: slope; q: intercept; SE: standard error. Figure S4: Regression between AB (a.: AB1; b.: AB2; c.: AB3) and the gravimetric method (EPA WINS). Table S6: Regression parameters between direct-reading instruments (1-min averaged data). N: number of data; R: Pearson correlation coefficient; p: significance; m: slope; q: intercept; SE: standard error. Table S7: Regression parameters between AB and EPA WINS (8-h averaged data). N: number of data; R: Pearson correlation coefficient; p: significance; m: slope; q: intercept; SE: standard error. Regression parameters were calculated and reported for the summer and winter datasets. Figure S5: Bland-Altman plot. Red dotted lines represent upper and lower confidence intervals (95%) while the green dotted line represents the average difference between instruments. The mean concentrations between EPA WINS and the compared instruments (a.: AB1; b.: AB2; c.: AB3) are reported on the x-axis while the differences between the methods are shown (8-h average) on the y-axis. Table S8: Relative error (%) calculated during all monitoring sessions between direct-reading instruments and the gravimetric method. Table S9 Absolute error (µg/m^3^) calculated during all monitoring sessions between direct-reading instruments and the gravimetric method. Figure S6: Analysis of absolute error (absolute value - µg/m^3^) for ABs as a function of PM_2.5_ concentrations (µg/m^3^) and RH (%). Figure S7: Analysis of relative error (absolute value - %) for ABs as a function of PM_2.5_ concentrations (µg/m^3^) and RH (%). Figure S8: Analysis of absolute error (absolute value - µg/m^3^) for direct-reading instruments as a function of PM_2.5_ concentrations (µg/m^3^) and RH (%). Figure S9: Analysis of relative error (absolute value - %) for direct-reading instruments as a function of PM_2.5_ concentrations (µg/m^3^) and RH (%). Figure S10 Analysis of relative error (%) for direct-reading instruments as a function of PM_2.5_ concentrations (µg/m^3^) and RH (%). a.: AB1; b.: AB2; c.: AB3.
