# Supplementary material for: Precision and Accuracy of a Direct-Reading Miniaturized Monitor in PM2.5 Exposure Assessment
Source: Sensors (Basel). 2018 Sep 13;18(9):3089. doi: 10.3390/s18093089 (PMC6164905; doi:10.3390/s18093089)
Supplement: Supplementary file 1 [file sensors-18-03089-s001.pdf]

Article

Supplementary Material

Precision and Accuracy of a Direct-Reading Miniaturized Monitor in $PM_{2.5}$ Exposure Assessment

Francesca Borghi [1,*], Andrea Spinazzè [1,*], Davide Campagnolo [1], Sabrina Rovelli [1,2], Andrea Cattaneo [1], Domenico M. Cavallo [1]

[1] Department of Science and High Technology, Università degli Studi dell'Insubria, via Valleggio 11, 22100 Como, Italy; davide.campagnolo@uninsubria.it (D.C.); sabrina.rovelli@uinsubria.it (S.R.); andrea.cattaneo@uninsubria.it (A.C.); domenico.cavallo@uninsubria.it (D.M.C.)

[2] Department of Statistics, Informatics and Applications "G. Parenti", Università degli Studi di Firenze, viale Morgagni 59, 50134 Firenze, Italy

* Correspondence: f.borghi2@uninsubria.it (F.B.); andrea.spinazze@uninsubria.it (A.S.); Tel.: +39-031-238-6629 (F.B; A.S.)

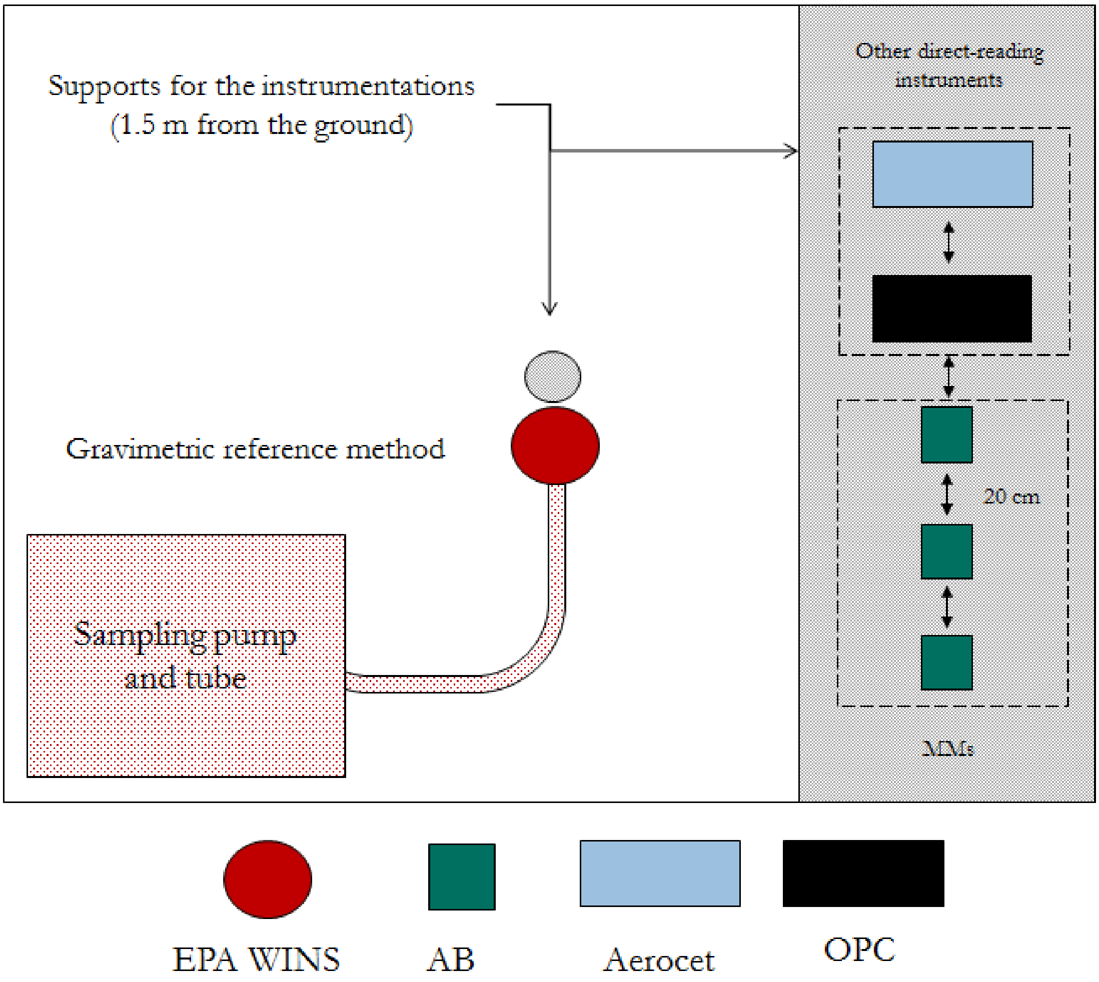

Figure S1. Setup of the sampling equipment and relative position (view from the above).

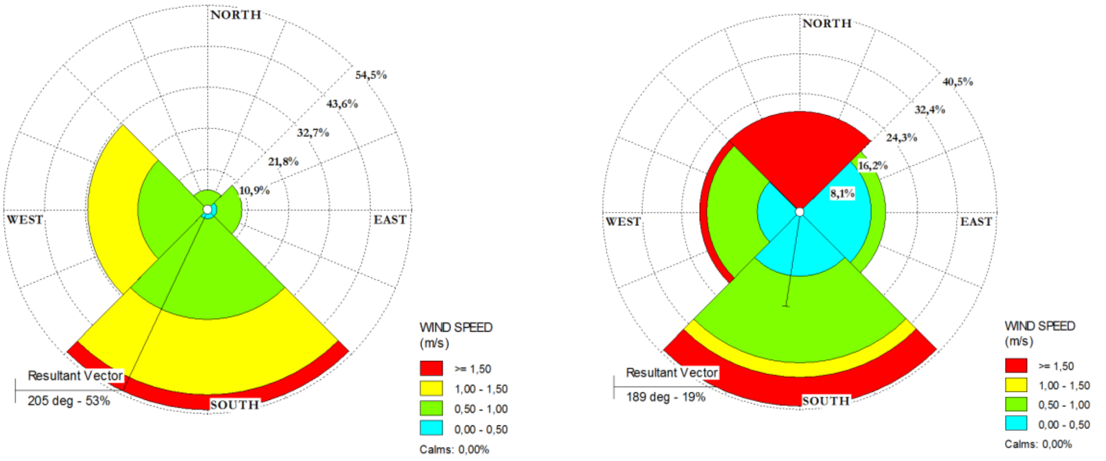

Figure S2. Wind direction (°) and intensity (m/s) during warm and cold periods.

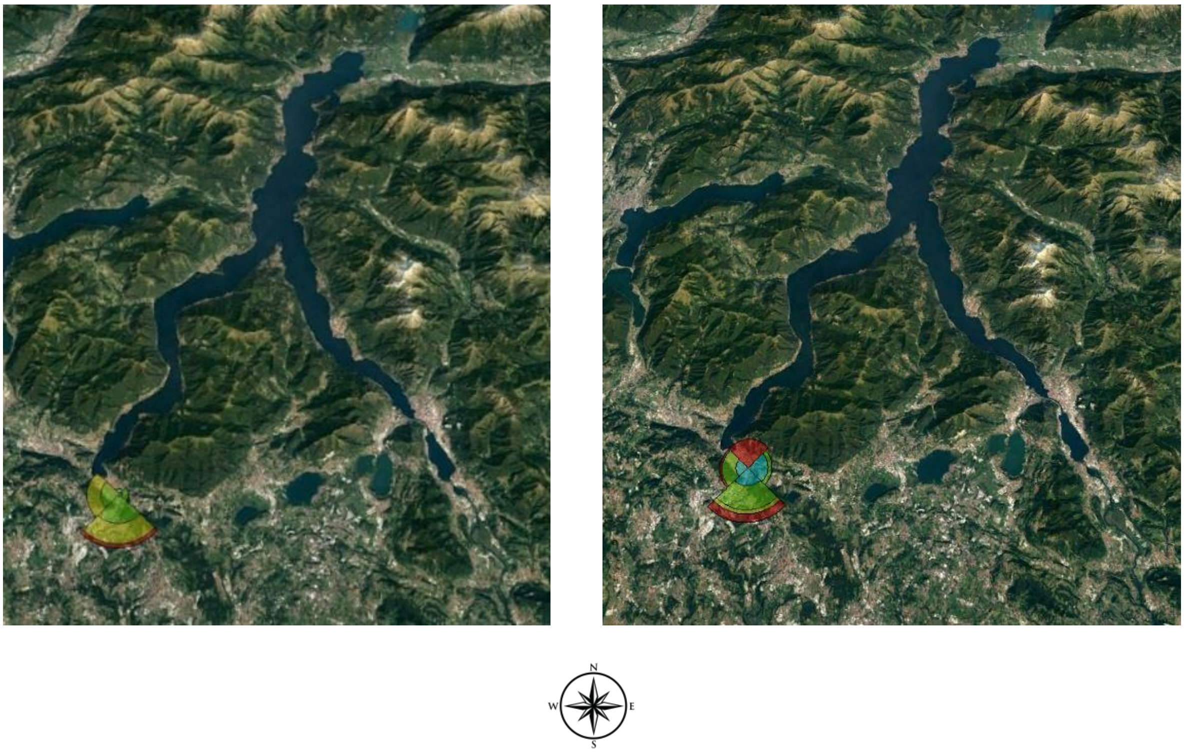

Figure S3. Maps of wind direction and intensity at the sampling point during cold and warm periods. Red areas correspond to wind intensity ≥ 1.50 m/s, yellow areas to wind intensity between 1 and 1.5 m/s, green areas between 0.5 and 1 m/s, and blue to wind intensity between 0 and 0.5 m/s.

Table S1. Error between ABs - descriptive statistic. S.D.: standard deviation; Max.: maximum; Min.: minimum; C.I.: confidence interval.

	ABx (relative error %)	ABx (abosulte error μg/m^3)
Mean	9	5.7
S.D.	64	15.5
Max	122	47.9
Min.	-70	-8.8
Range	192	56.7
C.I. (95%)	31	7.5

Table S2. Mann-Whitney test statistics. Z: Mann-Whitney test statistics; Asymp. Sig: significance.

	Mann-Whitney U	Z	Asymp. Sig. (2-tailed)
AB1 vs EPA WINS	168	-0.365	0.715
AB2 vs EPA WINS	170	-0.307	0.759
AB3 vs EPA WINS	165	-0.453	0.651
Aerocet vs EPA WINS	154	-0.774	0.439
OPC vs EPA WINS	167	-0.394	0.693

Table S3. Correlations between all ABs (8-h averaged data). All correlations are significant at 0.001 level and results are based on 19 monitoring sessions. In the table is reported the Spearman's rank order correlation (rho).

	EPA WINS	AB1	AB2	AB3	Aerocet	OPC
AB1	0.954	---	0.939	0.947	0.979	0.884
AB2	0.889	0.939	---	0.988	0.981	0.963
AB3	0.881	0.947	0.988	---	0.982	0.935

Table S4. Correlations between direct-reading instruments (1-min averaged data). All correlations are significant at 0.001 level. In brackets are reported the number of data used for analysis. In the table is reported the Spearman's rank order correlation (rho).

	AB1	AB2	AB3	Aerocet	OPC
AB1	---	0.993 (N=6188)	0.989 (N=5862)	0.981 (N=7401)	0.986 (N=6813)
AB2	---	---	0.991 (N=5761)	0.983 (N=7241)	0.986 (N=6951)
AB3	---	---	---	0.978 (N=6851)	0.982 (N=6401)

Table S5. Regression parameters between direct-reading instruments (8-h average) and the gravimetric method. N: number of data; R: Pearson correlation coefficient; p: significance; m: slope; q: intercept; SE: standard error.

Instrument compared	Regression model				Slope			Intercept		
	N	R	R^2	*p*	m	SE	*p*	q	SE	*p*
AB1 vs EPA WINS	19	0.922	0.85	<0.001	1.375	0.14	<0.001	-0.473	0.17	0.013
AB2 vs EPA WINS	19	0.89	0.792	<0.001	1.346	0.168	<0.001	-0.416	0.203	0.056
AB3 vs EPA WINS	19	0.889	0.79	<0.001	1.272	0.159	<0.001	-0.347	0.193	0.09

	Comparable and mutually predictable	Comparable but ton mutually predictable
AB1 vs EPA WINS		YES
AB2 vs EPA WINS	NO	NO
AB3 vs EPA WINS		NO

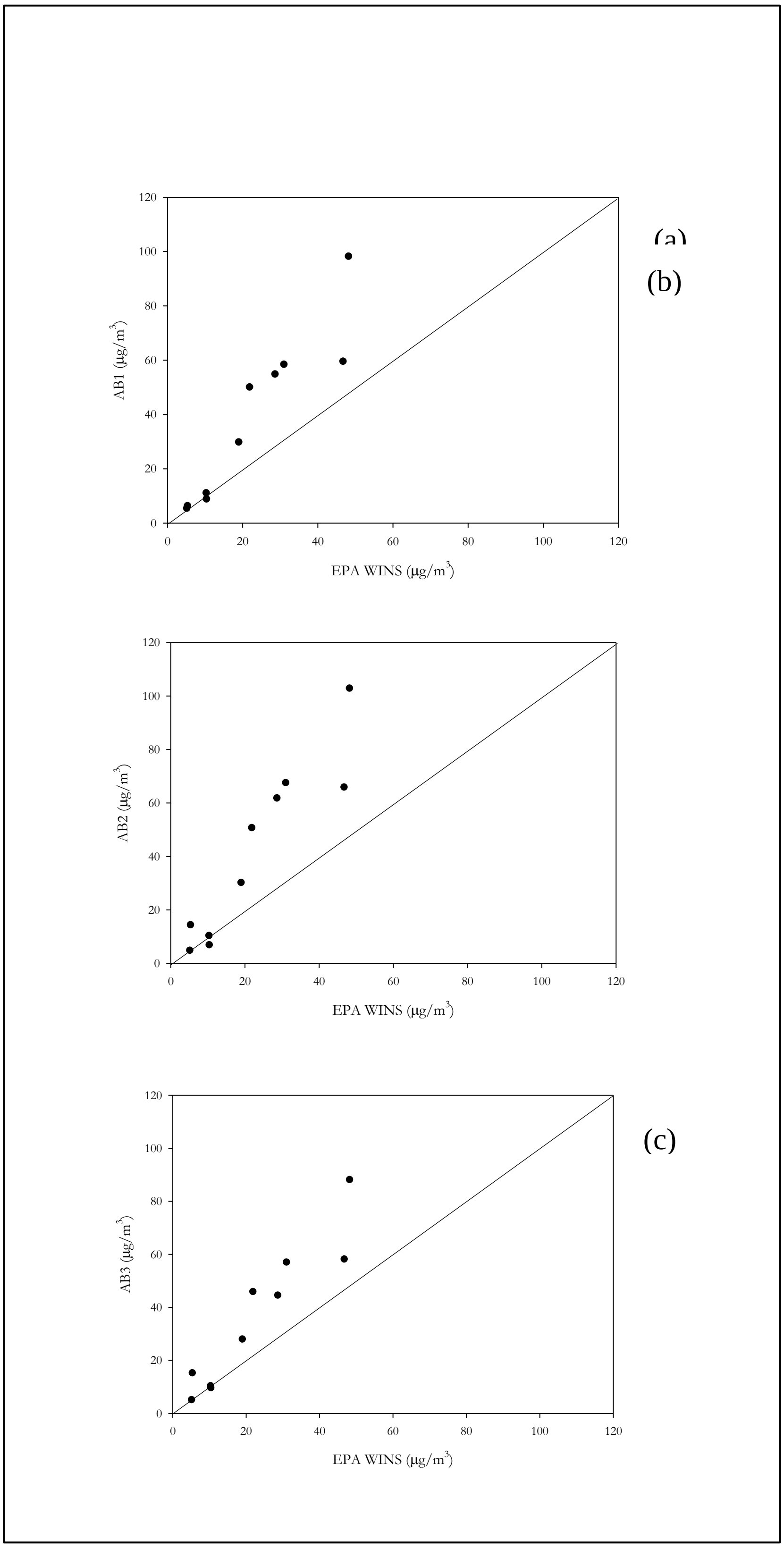
(a)
(b)
(c)
AB1 (μg/m3)
AB2 (μg/m3)
AB3 (μg/m3)
EPA WINS (μg/m3)
0
20
40
60
80
100
120

Figure S4. Regression between AB (a.: AB1; b.: AB2; c.: AB3) and the gravimetric method (EPA WINS).

Table S6. Regression parameters between direct-reading instruments (1-min averaged data). N: number of data; R: Pearson correlation coefficient; p: significance; m: slope; q: intercept; SE: standard error.

Instrument Compared	Regression model				Slope			Intercept			Comparable and mutually predictable	Comparable but not mutually predictable
	N	R	R^2	*p*	m	SE	*p*	q	SE	*p*		
AB1 vs Aerocet	7401	0.969	0.939	<0.001	0.933	0.003	<0.001	-0.063	0.004	<0.001		
AB2 vs Aerocet	7241	0.968	0.937	<0.001	0.963	0.003	<0.001	-0.096	0.004	<0.001	NO	YES
AB3 vs Areocet	6851	0.969	0.939	<0.001	0.944	0.003	<0.001	-0.073	0.004	<0.001		
AB1 vs OPC	6813	0.981	0.962	<0.001	0.878	0.002	<0.001	0.123	0.003	<0.001		
AB2 vs OPC	6851	0.969	0.939	<0.001	0.944	0.003	<0.001	-0.073	0.004	<0.001	NO	YES
AB3 vs OPC	6401	0.979	0.958	<0.001	0.881	0.002	<0.001	0.124	0.003	<0.001		

Table S7. Regression parameters between AB and EPA WINS (8-h averaged data). N: number of data; R: Pearson correlation coefficient; p: significance; m: slope; q: intercept; SE: standard error. Regression parameters were calculated and reported for the summer and winter datasets.

Summer Database

Instrument compared	Regression model				Slope			Intercept		
	N	R	R^2	*p*	m	SE	*p*	q	SE	*p*
AB1 vs EPA WINS	9	0.980	0.960	<0.001	0.361	0.049	<0.001	-0.865	0.698	0.255
AB2 vs EPA WINS	9	0.982	0.964	<0.001	0.621	0.045	<0.001	-0.627	0.646	0.364
AB3 vs EPA WINS	9	0.986	0.972	<0.001	0.633	0.040	<0.001	-0.911	0.572	0.155

Winter Database

Instrument compared	Regression model				Slope			Intercept		
	N	R	R^2	*p*	m	SE	*p*	q	SE	*p*
AB1 vs EPA WINS	10	0.940	0.884	<0.001	1.839	0.235	<0.001	-3.816	6.414	0.568
AB2 vs EPA WINS	10	0.940	0.884	<0.001	1.967	0.253	<0.001	-3.432	6.915	0.633
AB3 vs EPA WINS	10	0.944	0.891	<0.001	1.614	0.200	<0.001	-0.763	5.450	0.892

.

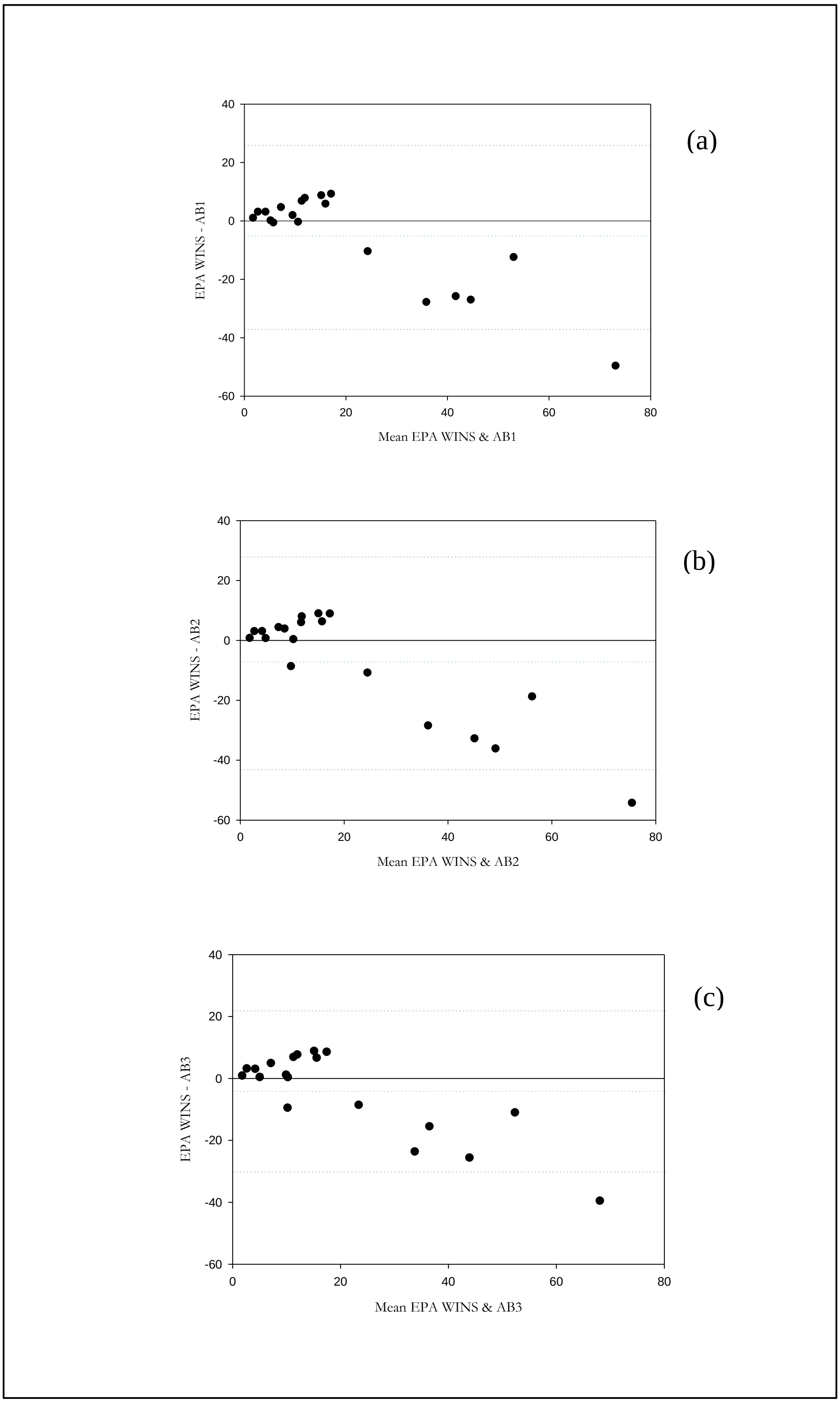

Figure S5. Bland-Altman plot. Red dotted lines represent upper and lower confidence intervals (95%) while the green dotted line represents the average difference between instruments. The mean concentrations between EPA WINS and the compared instruments (a.: AB1; b.: AB2; c.: AB3) are

reported on the x-axis while the differences between the methods are shown (8-h average) on the y-axis.

Table S8. Relative error (%) calculated during all monitoring sessions between direct-reading instruments and the gravimetric method.

Session	Season	Relative Error					
		ABx	AB1	AB2	AB3	OPC	Aerocet
2	Summer	-70.0%	-70.2%	-68.5%	-71.8%	-81.0%	-67.0%
3	Summer	-34.0%	-39.6%	-29.8%	-33.8%	-60.0%	-22.0%
4	Summer	-51.0%	-51.9%	-50.9%	-51.0%	-62.0%	-27.0%
5	Summer	-44.0%	-45.5%	-39.9%	-45.9%	-49.0%	-5.0%
6	Summer	-47.0%	-47.7%	-44.2%	-50.1%	-46.0%	-6.0%
7	Summer	-49.0%	-48.6%	-49.6 %	-47.6%	-50.0%	-6.0%
8	Summer	-45.0%	-44.1%	-45.2%	-44.5%	-48.0%	-3.0%
9	Summer	-40.0%	-42.0%	-40.4%	-38.8%	-40.0%	0.0%
10	Summer	-32.0%	-30.2%	-32.6%	-34.4%	-27.0%	50.0%
11	Winter	-21.0%	-17.4%	-35.8%	-9.8%	n.a	38.0%
12	Winter	0.0%	4.5%	-2.4%	-2.3%	9.0%	48.0%
13	Winter	95.0%	87.2%	116.5%	82.7%	152.0%	121.0%
14	Winter	117.0%	12.9%	160.8%	176.2%	204.0%	245.0%
15	Winter	-6.0%	-0.5%	-11.3%	-5.9%	3.0%	32.0%
16	Winter	30.0%	26.8%	40.3%	23.8%	42.0%	49.0%
17	Winter	86.0%	90.2%	114.4%	54.4%	147.0%	125.0%
18	Winter	99.0%	102.9%	112.5%	82.1%	177.0%	206.0%
19	Winter	53.0%	55.1%	57.2%	45.6%	166.0%	147.0%
20	Winter	122.0%	127.1%	130.0%	108.2%	97.0%	121.0%

Table S9. Absolute error ($\mu g/m^3$) calculated during all monitoring sessions between direct-reading instruments and the gravimetric method.

Session	Season	Absolute error					
		ABx	AB1	AB2	AB3	OPC	Aerocet
2	Summer	-2.99	-2.99	-2.92	-3.06	-3.45	-2.85
3	Summer	-0.77	-0.89	-0.67	-0.76	-1.34	-0.49
4	Summer	-2.95	-2.99	-2.93	-2.94	-3.57	-1.57
5	Summer	-6.46	-6.71	-5.89	-6.77	-7.20	-0.68
6	Summer	-4.54	-4.57	-4.24	-4.80	-4.42	-0.62
7	Summer	-7.72	-7.72	-7.87	-7.56	-7.93	-1.02
8	Summer	-8.72	-8.63	-8.84	-8.70	-9.35	-0.49
9	Summer	-8.78	-9.13	-8.79	-8.43	-8.75	-0.07
10	Summer	-6.14	-5.72	-6.18	-6.51	-5.17	9.53
11	Winter	-2.21	-1.83	-3.77	-1.03	-10.52	4.01

12	Winter	-0.01	0.47	-0.25	-0.24	0.96	5.06
13	Winter	29.71	27.14	36.26	25.74	47.33	37.72
14	Winter	6.37	0.70	8.79	9.63	11.14	13.39
15	Winter	-0.31	-0.03	-0.60	-0.31	0.16	1.70
16	Winter	14.19	12.54	18.88	11.15	19.68	22.80
17	Winter	24.82	25.93	32.90	15.63	42.30	36.00
18	Winter	47.93	49.73	54.39	39.67	85.57	99.43
19	Winter	10.06	10.53	10.94	8.71	31.83	28.09
20	Winter	26.76	27.92	28.58	23.78	21.38	26.60

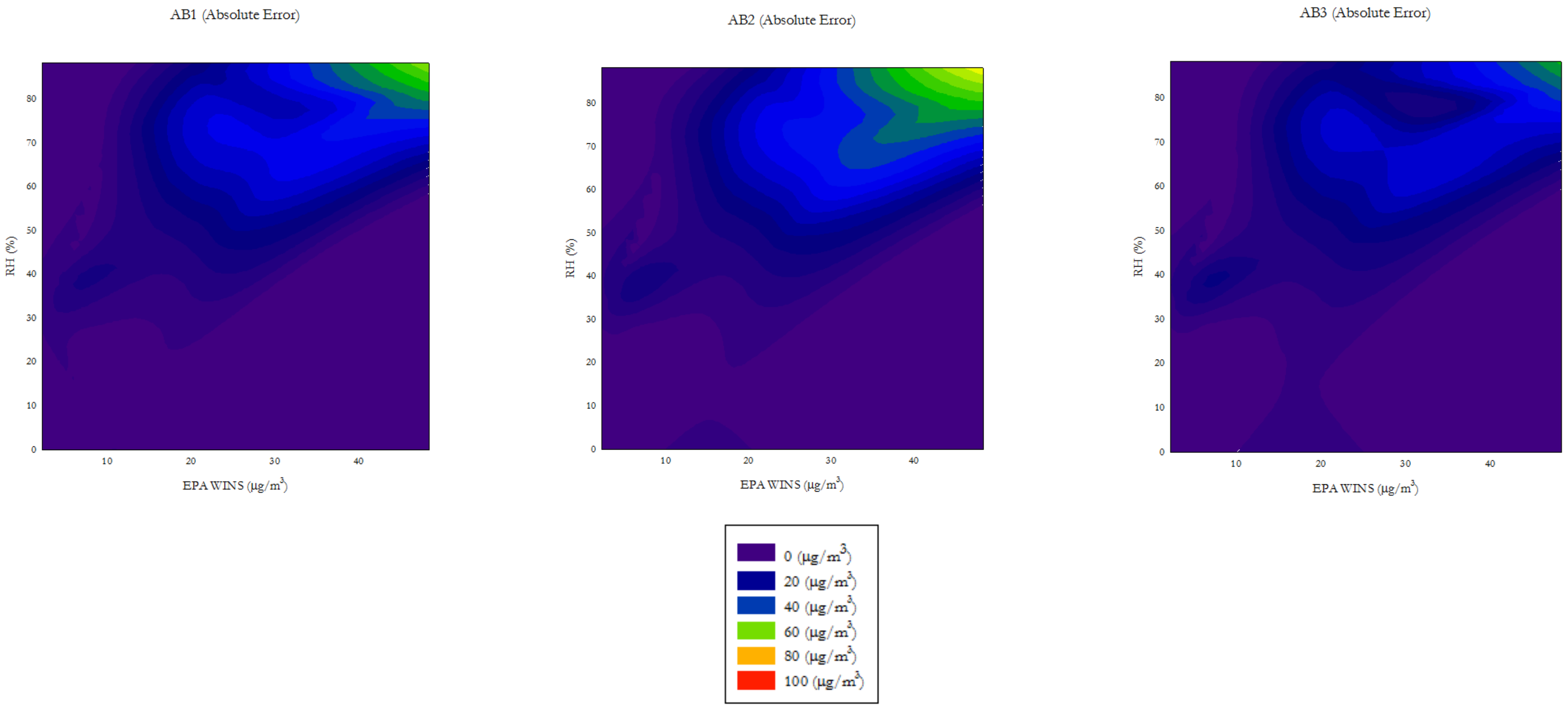

Figure S6. Analysis of absolute error (absolute value - µg/m^3) for ABs as a function of $PM_{2.5}$ concentrations (µg/m^3) and RH (%).

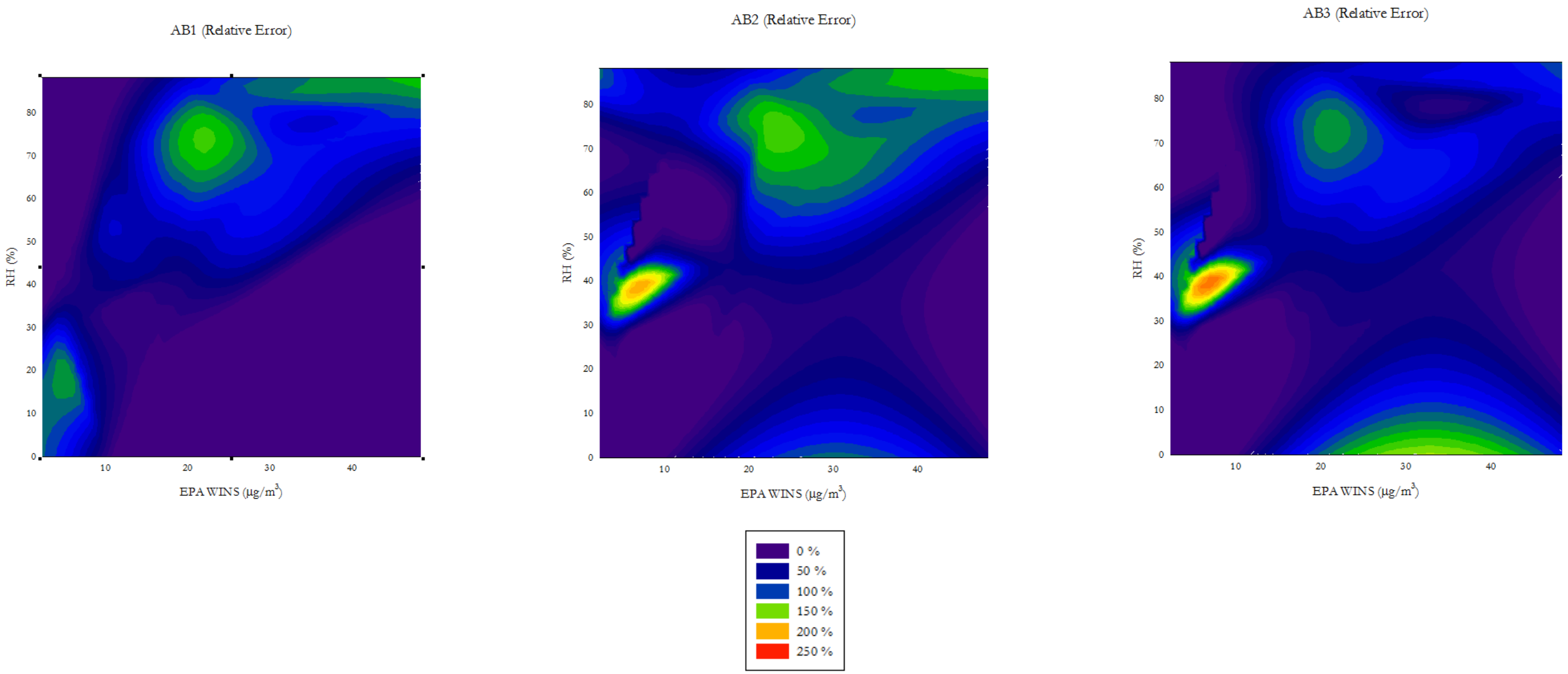

Figure S7. Analysis of relative error (absolute value - %) for ABs as a function of $PM_{2.5}$ concentrations (μg/m³) and RH (%).

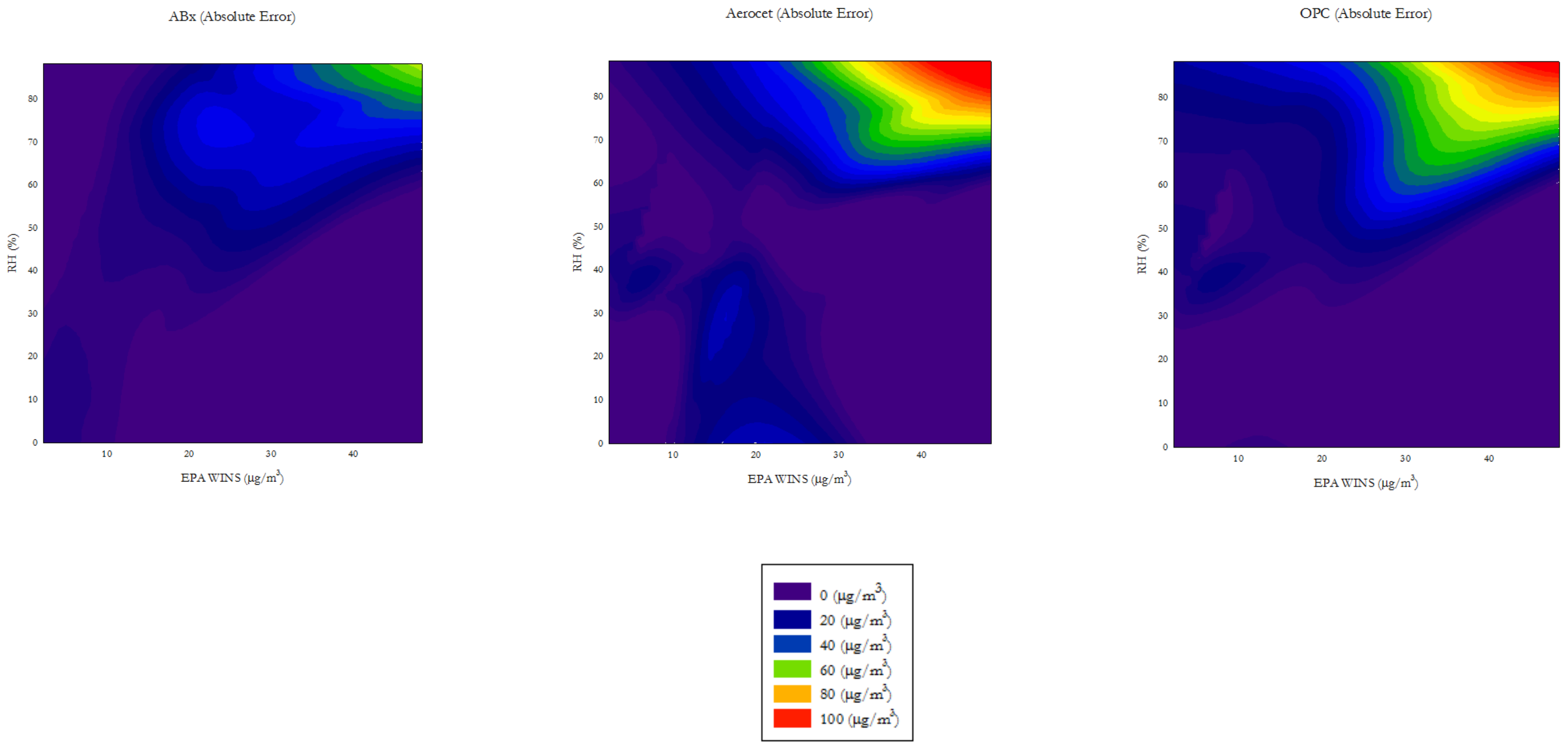

Figure S8. Analysis of absolute error (absolute value - μg/m³) for direct-reading instruments as a function of $PM_{2.5}$ concentrations (μg/m³) and RH (%).

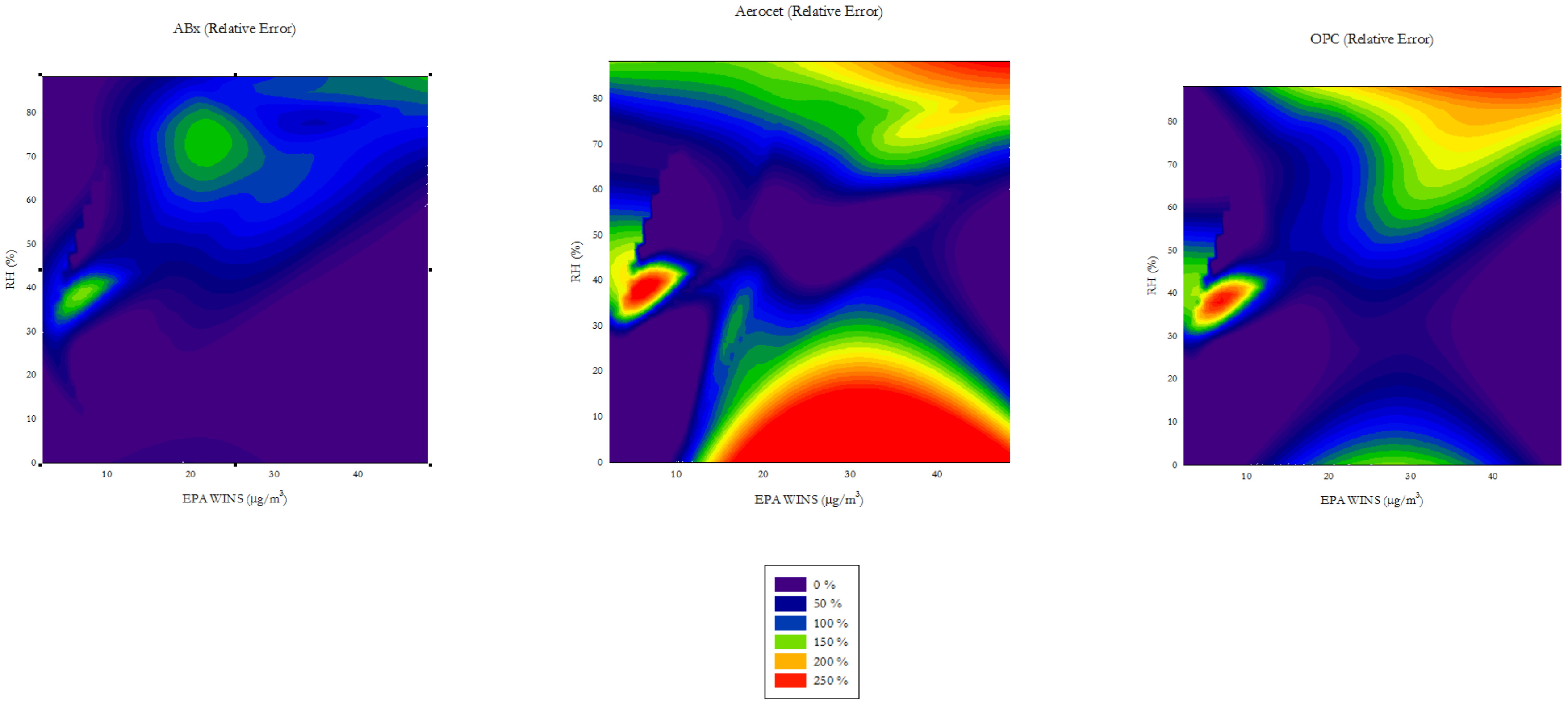

Figure S9. Analysis of relative error (absolute value - %) for direct-reading instruments as a function of $PM_{2.5}$ concentrations (µg/m^3) and RH (%).

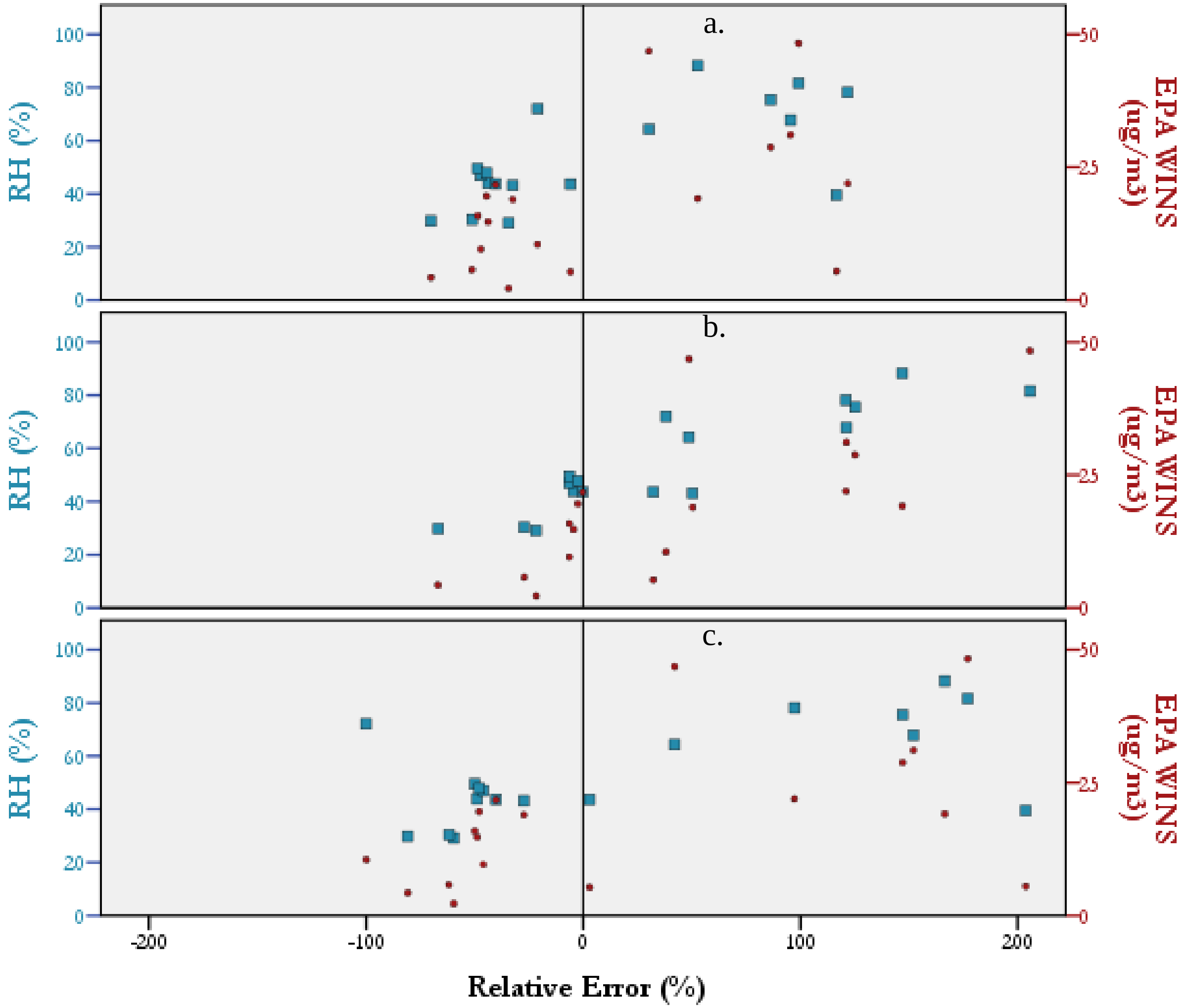

Figure S10. Analysis of relative error (%) for direct-reading instruments as a function of $PM_{2.5}$ concentrations (μg/m³) and RH (%). a.: AB1; b.: AB2; c.: AB3.
